# Identification of a HOMA-IR cut-off point for cardiometabolic risk and modifiable risk factors in peruvian adolescents

**DOI:** 10.1371/journal.pone.0351139

**Published:** 2026-07-10

**Authors:** Katherine Curi-Quinto, Fabian Vasquez, Melissa Abad, Fabiola Lazarte, Mary Penny, Juana del Valle-Mendoza

**Affiliations:** 1 Research Center of the Faculty of Health Sciences, Universidad Peruana de Ciencias Aplicadas, Lima, Peru; 2 Nutritional Research Institute, Lima, Peru; 3 Finis Terrae University, Faculty of Medicine, School of Nutrition and Dietetic, Santiago, Chile; Instituto Nacional de Cardiologia Ignacio Chavez, MEXICO

## Abstract

**Background:**

Although HOMA-IR is widely used to assess insulin resistance, reported cut-off values vary substantially across population, particularly during adolescence. The aim of this study was to determine the distribution of HOMA-IR values. identify a HOMA-IR cut-off associated with metabolic syndrome (MS), and assess modifiable risk factors of IR in a longitudinal cohort of Peruvian adolescents.

**Methods:**

We performed a secondary data analysis from a longitudinal adolescent’s study. A sample of 371 adolescents (14.5 ± 0.1 years old) from low- medium socioeconomic status. ROC curve analysis was used to identify the specific cut-off point to classify IR using the sensitivity and specificity values in comparison with the MS. Multiple logistic regression analysis including diet, physical activity and body composition from adolescence, excess weight during infancy and family history of non-chronic disease was included to identify risk factors (FHCD) associated with IR.

**Results:**

The HOMA-IR was 3.29 (SD 1.71) with no differences by sex. We identified 3.9 for HOMA-IR as the cut-off point with sensitivity (72.4%) and specificity (75.4%) for predicting MS. IR was present in 28.6% (95% CI 24.2;33.4%); 84% had at least one cardiometabolic risk factor and low HDL and abdominal obesity were the most prevalent (62 and 35%, respectively). Adolescents with higher fat mass index (OR 16.03, 95% CI 6.79 to 37.86), and those physically inactive (OR 2.08 95% CI 1.06 to 4.07) were more likely to have IR. No association was found with diet, excess weight at infancy and FHCD.

**Conclusions:**

A cut-offs point of 3.9 for HOMA-IR allows to identify adolescents with high metabolic risk. Strategies to promote lower FMI and improve the physical activity levels could reduce the risk of IR in adolescents.

## Introduction

Early exposure to negative changes in lifestyle such as dietary patterns and physical activity have increased the risk for obesity and cardiometabolic disorders earlier in life [1–3]. According to the World Atlas of Obesity 2023, children and adolescents are the most vulnerable population because the prevalence of obesity in these groups is likely to increase from 2020 to 2030 by 10–17% in males and from 8% to 14% in females [4]. Along with obesity, there is an early onset of cardiometabolic disorders such as metabolic syndrome (MS). For instance, in adolescents the prevalence of MS was 5.5% (4.1–8.4) in high-income countries, 3.9% (3.1–5.4) in upper-middle-income countries, 4.5% (2.6–8.4) in lower-middle-income countries, and 7.0% (2.4–15.7) in low-income countries [5].

MS is a cluster of cardiometabolic disorders that includes abdominal obesity, glucose intolerance, hypertension, and dyslipidemia [6]. Insulin resistance (IR) has been considered an underlying factor of MS, and this is defined as the reduction in the tissue response to insulin stimulation, causing impaired glucose uptake by cells, and increased circulating glucose (hyperglycemia). Faced with hyperglycemia, the first response of cells is to increase circulating insulin levels, then cell metabolism changes to alternative pathways that cause metabolic alterations such as adipose tissue dysfunction, production of reactive oxidative species, inflammation, dyslipidemia, atherosclerosis, endothelial dysfunction, and hypertension [7]. Having these conditions earlier in life increases the risk for chronic noncommunicable diseases (NCDs) such as type 2 diabetes and cardiovascular disease in adulthood [8,9]. Therefore, early detection of IR, as well as the identification of its main risk factors at a country-specific level gives the opportunity to initiate actions to control the occurrence of NCDs in a timely manner.

Homeostasis model assessment-estimated insulin resistance (HOMA-IR) is the most common method used to measure IR [10,11]. HOMA-IR is a validated surrogate measure for IR that is calculated using data of fasting plasma glucose and insulin. This method was developed by Matthews *et al.* and this is a more accessible and non-invasive method compared to the gold standard of hyperinsulinemic euglycemic clamp [12,13]. HOMA-IR is affected by different factors in the study population, such as ethnicity, age, sex, and metabolic conditions; and it is therefore necessary to identify country specific HOMA-IR cut-off points for the classification of IR. Nonetheless, no studies in Peruvian adolescents have proposed specific cut-off points for HOMA-IR classification. Therefore, this study aimed to determine the distribution of HOMA-IR values and identify a cut-off value associated with MS, as well as identifying the modifiable risk factors of IR in a longitudinal study of Peruvian adolescents.

## Materials and methods

### Study design and population

This is a secondary analysis of data obtained from a population of Peruvian adolescents who were part of a longitudinal study that began at infancy (6–11 months of age) and was followed up with evaluations at 14 years of age. Participants recruited between 2004 and 2005 were healthy infants with birth weights higher than 2500 grams [14]. Infants were part of a trial designed to evaluate the efficacy of a complementary food based on bovine milk fat globule membranes (bMFGM) on diarrhea, anemia, and micronutrient status [14]. The follow-up phase was carried out in 2018 with the aim of evaluating the long-term effects of the use of bMFGM on health and nutritional outcomes at 14 years of age that showed no difference in body composition and cardiometabolic indicators among adolescents that was supplemented with bMFGM at infancy and the control group. From a total of 394 adolescents, we analyzed data of 371 participants that have complete data from infancy and adolescence. For fat-free mass (FFM) and fat-free mass index (FFMI), data were available for 349 participants; thus, related bivariate analyses used this subsample, while others used the full sample (n = 371).

The study has been approved by the Ethics Committee of the Instituto de Investigación Nutricional (Lima, Peru). Parents or caregivers of the participants signed an informed consent form and adolescents assented to participation.

### Adolescence nutritional status

Nutritional status was assessed using the z-score of body mass index for age that was calculated based on anthropometric measures of weight and height. Standardized procedures were used for these measurements. Weight was measured with a SECA scale with a precision of 0.1 kg; height was measured with a Holstein stadiometer with 0.1 cm accuracy. For anthropometric assessment World Health Organization’s reference standards (2007) were used. Standardized for age and sex body max index (BMI) was used as an indicator of overweight. Values of BMI z score >2 standard deviation (SD) were classified as obesity, values >1SD were considered overweight and values  < 2SD were underweight.

### Adolescent cardiometabolic risk factors

Waist circumference (WC) defined as the minimum circumference between the iliac crest and the rib cage was measured using a distensible measuring tape (SECA). Fasting serum total glucose, insulin, and lipid profile (cholesterol, triglycerides, and high-density lipoprotein) were measured in venous blood sample obtained after 12 h of fasting using an enzymatic colorimetric test (QCA SA Amposta, Spain) and dry analytical methodology (Vitros, Johnson & Johnson Clinical Diagnostics Inc.), respectively. The systolic and diastolic blood pressures were measured using a standard mercury sphygmomanometer, on the non-dominant arm at rest on a level surface of the heart 15 min at rest.

### Adolescent body composition

Body composition was evaluated using bioimpedance measurement (Seca mBCA 525). Indicators of FMI and FFMI were estimated. FMI was calculated as total fat mass value divided by height squared, and FFM as the total value of fat free mass by height squared. FMI, FFMI were classified into tertiles (T1, T2, T3).

### Diet and physical activity

Quality of diet was assessed based on food intake using a food frequency questionnaire. Trained nutritionists asked about food frequency intake of seven predefined lists of critical food groups: meat and sausages, dairy products, legumes, vegetable, fruits, sweet sugar beverages, sweet and salty snacks, consumed in the last month before the interview. We defined the diet as relatively healthy or unhealthy whether a person fulfilled the recommended intake of at least four food groups [15]. Physical activity was measured using a 7-day recall questionnaire for adolescents (PAQ-A) that was applied by trained field workers who registered the answers of the adolescents. The PAQ-A was developed by Kowalski *et al*, 1997 and this is an accessible tool with a good content validity as well as moderate positive correlation with VO^2^ peak and cardiorespiratory fitness [16,17]. The PAQ-A was used in the Peruvian context [18] and this assessed the general level of physical activity through eight questions about physical activity that the adolescent carried out in the last 7 days during their free time, during physical education classes, at different times during class days (lunch, afternoons, and evenings) and during the weekend. Each item was scored on a 5-point scale. The final score was obtained by the arithmetic average of the scores obtained from these 8 questions. Question 9 informed about any circumstance that prevented him from doing physical activity that week and this factor was considered in the analysis.

### Nutritional status at infancy and family history of chronic diseases

Weight and length/ height data for the first and second year of age were included. Anthropometric measures were assessed by trained personnel following standardized procedures [19]. The WHO reference standards were used to obtain z-score for weight, length/height and BMI for age. Childhood overweight and obesity was diagnosed when the z-BMI/age was > 2 SD and >3 SD, respectively. Excess weight was defined as infants with overweight and obesity (z-BMI < 2 SD). Family history of chronic diseases was obtained from self-reports by parents or caregivers of adolescents. Presence or absence of a history of DM2, hypertension and obesity in parents and siblings were considered.

### Definition of Metabolic Syndrome

MS was diagnosed using the International Diabetes Federation (IDF-2007) [20] criteria that is established when a subject has altered waist circumference (WC > 90 cm in men and 80 cm in women); plus two risk factors that included: systolic blood pressure ≥130 or diastolic blood pressure ≥ 85 mmHg, triglycerides ≥ 150 mg / dl, high density lipoprotein (HDL) ≤ 40 mg / dl in men and in women ≤50; and fasting blood glucose ≥ 100 mg / dl.

### Definition of Insulin resistance

IR was estimated using the HOMA-IR, calculated as the product of fasting insulin (µU/mL) and fasting glucose (mmol/L), divided by 22.5. This index provides an indirect measure of insulin sensitivity based on fasting metabolic parameters [21]. Receiver operating characteristic (ROC) analysis was used to find the optimal cut-off of IR for MS diagnosis in Peruvian adolescents. A test with perfect discrimination has a ROC plot that passes through the upper left corner, indicating 100% sensitivity and 100% specificity. A ROC plot closer to the upper left corner denotes greater accuracy of the test. To determine optimal cutoffs for MS diagnosis, the point on the ROC curve with maximum Youden Index [sensitivity-(1-specificity)] was calculated. Next, the values were verified with the likelihood ratio for a positive result (LR+) and the post-test probability (the proportion of participants above cutoffs who truly have MS).

### Ethical considerations

The ethics committees of the Instituto de Investigación Nutricional (IIN), Lima-Peru, approved this study under the number 372–2017/CIEI-IIN. This study was conducted in accordance with the ethical principles of the Declaration of Helsinki. We obtained written informed consent from all participants prior to their inclusion as well as the assent from the adolescents.

### Statistical analysis

Quantitative variables were described using mean and standard deviation (SD) or confidence intervals. Categorical variables were described by absolute and relative frequencies. To test differences of means we used t-test for independent samples or chi-square to test the association among categorical variables. We estimated the bivariate association among potential risk factors and IR using bivariate binomial logistic regression. We used the multivariate logistics regression model to identify the risk factor of IR. Two models were tested. In the first model (model 3), we included potential risk factors from adolescence, and in the second model (model 4). We included early potential risk factors (excess weight at childhood and FHCD). For both models we included the adjustment for the participation of the trial at infancy. A P-value < 0·05 was considered as statistically significant, and all analyses were conducted by Stata software v.17.

## Results

**Fig 1:**
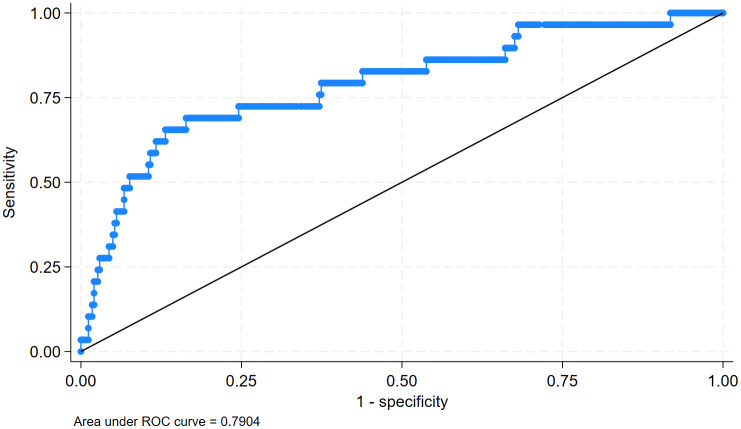
ROC curve to determine the optimal cutoff value of HOMA-IR for metabolic syndrome diagnosis in adolescents. Receiver Operating Characteristic (ROC) curve illustrating the diagnostic performance of HOMA-IR in identifying metabolic syndrome among adolescents. The area under the curve (AUC) is 0.7904, indicating good discriminatory power. The curve shows the trade-off between sensitivity and 1-specificity across a range of HOMA-IR cutoff values.

The HOMA-IR cutoff identified was 3.9, yielding a sensitivity of 72.4% and a specificity of 75.4%. The area under the ROC curve was 0.79 (95% CI: 0.69–0.88) (Fig 1). The prevalence of obesity and overweight in the adolescents was 14.6% (95% CI 11.3; 18.5%) and 27.5% (95% CI 23.2; 32.3%), respectively, and 48.1% of the population was female. Additionally, prevalence of MS according to the IDF criteria was 7.8% (95% CI 5.5; 11.0).

Table 1 shows that mean HOMA-IR was 3.3 (95% CI 3.1–3.5) and presents the distribution of HOMA-IR percentiles by sex, body mass index and MS. The mean HOMA-IR was significantly higher in overweight and obese adolescents compared with those with healthy weight (3.42; 5.17 vs 2.75), and those with MS compared with those without MS (5.31 vs 3.11). No significant difference was found by sex. Table 1 presents the optimal cutoff points for HOMA-IR to predict MS in males and females.

**Table 1:** Percentile distribution of the HOMA-IR in the overall sample and stratified by sex, body mass index and metabolic syndrome.

	Sample by sex			z-Score Body mass Index/Age			Metabolic Syndrome (IDF)^2^	
HOMA-IR^1^ Percentiles	Total (n = 371)	Male (n = 193)	Females (n = 178)	Healthy (≤ 1) n = 215	Overweight (1–1.99) n = 102	Obesity (≥2) n = 54	No (n = 342)	Yes (n = 29)
1%	0.81	0.76	1.09	0.81	1.29	1.83	0.81	1.54
5%	1.23	1.18	1.54	1.17	1.65	2.28	1.23	2.21
10%	1.63	1.51	1.75	1.36	1.73	2.47	1.59	2.24
25%	2.09	1.99	2.21	1.91	2.28	3.25	2.02	3.26
50%	2.83	2.64	3.04	2.47	3.12	4.98	2.73	5.56
75%	4.01	4.10	3.97	3.48	4.08	6.79	3.90	7.05
90%	5.85	6.27	5.17	4.31	5.29	7.60	5.12	8.37
95%	7.04	7.25	6.37	5.17	6.54	8.90	6.35	8.41
99%	8.90	9.74	7.77	6.30	8.45	9.79	8.45	9.79
Mean	3.29	3.30	3.27	2.75	3.42 ^a^	5.17 ^ab^	3.11	5.31 ^c^
SD	1.71	1.91	1.45	1.21	1.67	2.06	1.55	2.16

^1^Homeostasis model assessment-estimated insulin resistance (fasting insulin (μU/mL) x fasting glucose (nmol/L)/22.5)

^2^Defined by the International Diabetes Federation (IDF) criteria:

Central obesity (WC waist circumference >90 cm in males and 80 cm in females), plus two metabolic risk factors.

^3^Standard Deviation

^a^Indicates a statistically significant difference compared with the healthy body mass index category (ANOVA). ^b^ Indicates a statistically significant difference compared with the overweight category (ANOVA). ^c^ Indicates a statistically significant difference between participants with and without metabolic syndrome (t-test). Differences were considered statistically significant at p < 0.05.

**Fig 2:**
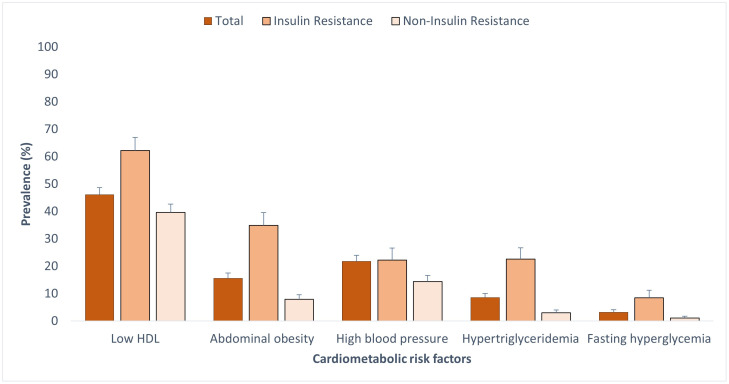
Mean prevalence of cardiometabolic risk factors by the presence of insulin resistance based on HOMA-IR values. Error bars: standard errors. Statistically significant differences by Pearson Chi^2^, comparing IR with non-IR. Abbreviations: SM is defined as abdominal obesity (> 90 cm for men and 80 cm for women) plus two of the following risk factors: SBP ≥ 130 or DBP ≥ 85 mmHg; Triglycerides ≥ 150 mg / dl; HDL ≤ 40 mg / dl in men and ≤50 in women; fasting glycaemia ≥ 100 mg / dl. *Indicates statistically significant differences among prevalences of risk factors among those with IR and those non-I.

To show differences in cardiometabolic risk factors by IR, Fig 2 presents the percentage distribution of each metabolic syndrome component. In the overall population at least six out of ten adolescents had at least one cardiometabolic risk factor (62%). As expected, in adolescents with IR eight out of ten (84%) had one or more cardiometabolic risk factors while in those classified as non-IR five out of ten (53%) had one or more cardiometabolic risk factors. The most prevalent risk factor in the overall sample as well as the adolescents with or without IR was the low levels of HDL (62.3% and 39.6%, respectively). Abdominal obesity and hypertriglyceridemia were the second and third most prevalent risk factors in adolescents with IR (34% and 22.6%, respectively) and about 8.5% had fasting hyperglycemia. The prevalence of all the cardiometabolic risk factors was significantly higher in adolescents with IR compared with those without IR (p < 0.001), except for hypertension in which the difference was not statistically significant (Fig 2).

Table 2 displays the anthropometric and cardiometabolic adolescent profile by IR status. Participants with IR compared with those without IR had significantly higher values of z-score for BMI/age, body composition indicators (FFMI and FMI) and cardiometabolic indicators including waist circumference, triglycerides, glucose, blood pressure, and insulin; while the HDL was significantly lower. Among those with or without IR, we did not find significant differences in the anthropometric profile from infancy including the z-score for height/age and body mass index/age, as well as the z-score for height/age in adolescence (Table 2).

**Table 2:** Anthropometric and cardiometabolic adolescent profile by insulin resistance.

			Insulin resistance based HOMA-IR^1^				
Variable	Total (n = 371)		IR (-) (n = 265)		IR (+) (n = 106)		*P* value^2^
	Mean	SD	Mean	SD	Mean	SD	
**Anthropometric profile at adolescence**							
Age (y)	14.5	0.1	14.5	0.1	14.5	0.1	0.171
Body mass index (Kg/m^2^)	22.6	3.9	21.6	3.2	25.1	4.4	<0.001
Height/Age (z-score)	−0.6	0.8	−0.6	0.8	−0.6	0.8	0.511
Body mass index/Age 14 y (z-score)	0.8	1.0	0.5	0.9	1.4	1.0	<0.001
**Body composition at adolescence** ^3^							
Fat Free Mass Index (FFMI)	16.6	2.4	16.4	2.4	17.2	2.2	0.007
Fat Mass Index (FMI)	5.9	3.9	5.2	3.8	7.8	3.3	<0.001
**Anthropometric profile at infancy**							
Height/Age at 8 months (z-score)	−0.6	0.9	−0.6	0.9	−0.5	0.9	0.809
Height/Age at 14 months (z-score)	−0.5	1.0	−0.5	1.0	−0.5	1.1	0.948
Body mass index/Age 8 months (z-score)	1.0	0.9	1.0	0.9	1.1	0.9	0.332
Body mass index/Age at 14 months (z-score)	0.9	0.9	0.8	0.9	0.9	1.0	0.358
**Cardiometabolic profile at adolescence**							
Waist circumference (cm)	75.5	9.3	73.0	7.3	81.6	10.8	<0.001
Triglycerides (mg/dl)	89.0	42.8	80.0	30.4	111.5	58.4	<0.001
High density lipoprotein (mg/dl)	46.7	11.6	48.5	11.1	42.1	11.6	<0.001
Glucose (mg/dl)	88.8	6.4	87.5	5.7	92.2	6.8	<0.001
Systolic blood pressure (mm Hg)	117.6	11.4	115.9	10.6	121.9	12.4	0.001
Diastolic blood pressure (mm Hg)	74.6	8.4	73.7	8.0	76.9	8.9	<0.001
Insulin (μIU/ml)	16.9	13.4	11.2	3.4	24.1	6.2	<0.001
HOMA-IR	3.3	1.7	2.4	0.7	5.5	1.5	<0.001

^1^Participants with values of HOMA-IR > 3.9 are classified as insulin resistance

^2^p-value from t-test for quantitative values and chi^2^ for the crude analysis.

^3^n=349

Table 3 presents the results of the bivariate analysis of potential risk factors for IR. Low levels of physical activity, overweight and obesity in adolescence, FMI, FFMI and presence of excess weight were significantly associated with the presence of MS. No significant association was found between diet, excess weight in the second year of age, and FHCD.

**Table 3:** Potential risk factors of insulin resistance in adolescents.

Risk factors	Adolescents with IR^1^		Adolescents without IR		OR^2^	P value^*^	CI 95%^3^
	N	%	N	%			
***Sex***							
Female	49	46.23	129	48.68	Ref.		
Male	57	53.77	136	51.32	1.10	0.669	0.70-1.73
***Food Intake***							
Healthy diet	69	65.09	182	68.68	Ref.		
Unhealthy diet^4^	37	34.91	83	31.32	1.18	0.505	0.73-1.89
***Low physical Activity***							
No	20	18.87	76	28.68	Ref.		
Yes^5^	86	81.13	189	71.32	1.73	0.053	0.99-3.01
***Nutritional status at 14 y***							
Normal weight	38	35.85	177	66.79	Ref.		
Overweight^6^	30	28.3	72	27.17	1.94	0.018	1.12-3.37
Obesity^7^	38	35.85	16	6.04	11.06	<0.001	5.60-21.86
**Body composition**							
**Fat free mass Index (kg/height**^**2**^)							
Low	29	29.29	92	36.8	Ref.		
Middle	30	30.3	88	35.2	1.09	0.765	0.60-1.95
High	40	40.4	70	28.0	1.81	0.041	1.02-3.21
**Fat mass Index (kg/height**^**2**^)							
Low	14	14.14	108	43.2	Ref.		
Middle	33	33.33	88	35.2	2.89	0.002	0.60-1.95
High	52	52.53	54	21.6	7.43	<0.001	1.02-3.21
***Excess weight during first year of age*** ^8^							
No	84	79.25	232	87.55	Ref.		
Yes	22	20.75	33	12.45	1.84	0.044	1.02-3.34
***Excess weight during second year of age*** ^8^							
No	93	87.74	235	88.68	Ref.		
Yes	13	12.26	30	11.32	1.09	0.798	0.55-2.19
***Family history of NCDs*** ^***9***^							
No	86	81	207	78	Ref		
Yes	20	19	58	22	0.83	0.520	0.47-1.46

^1^IR: Insulin resistance defined as HOMA-IR > 3.9; 2 OR: Odds Ratio; 3CI: Confidence Interval came from simple binomial logistic regression; 4Unhealthy diet: score ≤4; 5Physical inactivity: score <3; 6Overweight at 14y: z-BMI/age >1 Standard Deviation (SD); 7Obesity: z-BMI/age >2 SD. 8z-BMI ≥2 SD; 9 NCDs: non-communicable chronic disease includes diabetes, hypertension and obesity in parents or siblings. *Statistical significance p value < 0.05 from bivariate logistic regression model.

In the adjusted analysis using multiple logistic regression models (Table 4), low physical activity and high FMI were independently associated with IR. Those with low physical activity were two times more likely to have IR (OR: 2.08; 95% CI 1.06 to 4.07), and those with high FMI are more likely to have IR (OR: 5.38; 95% CI 2.41 to 11.99 for those in the tertile 2; and OR: 16.03; 95% CI 6.79 to 37.86 for those with high FMI compared with those with low FMI). These associations remained after adjusting for early factors in Model 2 including excess weight at infancy, FHNCD and the participation in the trial using the bMFGM in complementary food at infancy. None of this variable has been associated with the IR.

**Table 4:** Multivariate regression model of the associated risk factors of insulin resistance in Peruvian Adolescents.

Risk factors	Model 1		Model 2	
	OR	CI 95%	OR	CI 95%
**Female**	2.78*	1.17 - 6.62	2.89*	1.20 - 6.91
**Unhealthy diet** ^1^	1.01	0.58 - 1.76	0.97	0.55 - 1.71
**Low physical activity** ^2^	2.09*	1.07 - 4.07	2.08*	1.06 - 4.07
**Body composition**				
**Fat free mass Index (kg/height**^**2**^)				
Middle (Tertile 2)	1.20	0.60 - 2.40	1.16	0.57 - 2.35
High (Tertile 3)	1.53	0.61 - 3.84	1.48	0.58 - 3.74
**Fat mass Index (kg/height**^**2**^)				
Middle (Tertile 2)	5.15*	2.34 - 11.49	5.38*	2.41 - 11.99
High (Tertile 3)	14.96*	6.46 - 34.61	16.03*	6.79 - 37.86
**Excess weight at first year of age** ^3^	(…)	(…)	1.04	0.52 - 2.11
**Excess weight at second year of age** ^3^	(…)	(…)	1.07	0.49 - 2.36
**Familiar history of NCD** ^4^	(…)	(…)	0.61	0.31 −1.20
**Participation in the trial at infancy**	(…)	(…)	1.16	0.69–1.96

*Statistical significance p_value < 0.05 from multivariate logistic regression model; (...) Un-observed variable in the model, ^1^Unhealthy diet: score ≤4; ^2^Low physical activity: score <3; ^3^Excess weight z-BMI/age > 2 SD ^4^NCDs: non-communicable chronic diseases include diabetes, hypertension, and obesity in parents or siblings.

## Discussion

In this study we identified a value of 3.90 as specific cut-off point for HOMA-IR that has a sensitivity of 72.4% and specificity of 75.4% for predicting MS in Peruvian adolescents with an average age of 14.5 years (AUC: 0.79; 95% CI 0.69–0.88).

According to this cut-off point, 3 out of 10 adolescents had IR, and consistent with previous studies [22–24] this population had higher cardiometabolic risk factors such as low HDL (62%), abdominal obesity (35%), hypertriglyceridemia (23%), fasting hyperglycemia (8.5%), and MS (20%). This clustering of metabolic abnormalities reflects the central role of insulin resistance as a pathophysiological driver of cardiometabolic disease. Insulin resistance has been widely described as a key mechanism underlying metabolic syndrome, type 2 diabetes, and cardiovascular disease through its effects on glucose metabolism, lipid homeostasis, and systemic inflammation [25].

The identified cut-off point is close to previous studies in adolescents from 10 to 18 years old that reports a range of 2.50 to 3.29 for HOMA-IR for MS with values of AUC from 0.73 to 0.89 [26,27]. These findings are consistent with growing evidence supporting the role of HOMA-IR as an early marker of metabolic dysfunction beyond overt diabetes. In a large prospective cohort, demonstrated that elevated HOMA-IR levels independently predicted the development of type 2 diabetes and chronic kidney disease even in non-diabetic individuals, reinforcing its value as an early risk stratification tool [28]. As expected, this variability is associated with the different characteristic of the study populations coming from different countries (Korea, India, and Mexico), and have different cardiometabolic profile that can be noted by the different proportion for MS in each locations (1.6% in Korea to 19.9% in India), whereas in the Peruvian longitudinal study we found a prevalence of 7.8%, using the same criteria of IDF for the definition of MS). Despite this variability, HOMA-IR was recognized as a good alternative for detecting a population with high cardiometabolic risk early in life. The association and predictive capacity of adiponectin and HOMA-IR indexes with metabolic risk markers in 691 children and adolescents (7–14 years old); in both sexes HOMA-IR was associated with metabolic risk, and it was the most suitable methods for MS screening in both age groups [29]. Additionally, evidence from Peruvian populations suggests that insulin resistance is already elevated in individuals with prediabetes phenotypes and is associated with early metabolic alterations such as dyslipidemia and hepatic steatosis, even before the onset of overt diabetes [30].

In this study we also identified the low physical activity and higher FMI as independent risk factors for IR. In the case of physical activity, growing evidence recognizes its role as a factor for improving insulin sensitivity and prevention of metabolic disorders in young people. For instance, in a recent systematic review eleven of 16 studies suggested an independent association of physical activity level with metabolic disorders [31]. In relation to FMI, this is a measure of total body fat adjusted by the body size [FM (kg)/height (m)^2^]. Height is positively correlated with weight and this adjustment [32] removes this effect. For this reason, FMI is considered a better indicator than the relative value of body fat percentage [33]. Further, previous evidence shows that FMI compared with BMI and percentage of body fat (BF%) have a higher capacity for predicting MS [34]. In agreement with our results, previous studies that measured obesity by BMI as well as body fat have been positively associated with HOMA-IR, MS as well as cardiovascular diseases [23]. Excess of adipose tissue in obesity produces IR and increased release of free fatty acids in plasma; this is correlated with the magnitude and prevalence of abnormalities associated with IR, such as dyslipidemia, systemic inflammation, diabetes mellitus 2 hypertension, myocardial infarction, and early mortality. [35–38]

In this study we did not find an independent association between FFMI and HOMA-IR. We observed that adolescents in the high tertiles of FFMI compared with the low tertiles were most likely to have IR (1.81; 95% CI 1.02–3.21); however, this association was attenuated in the adjusted analysis mainly by the effect of the FMI, showing greater importance of the role of adipose tissue in relation to IR. These results are also in line with previous findings that report controversies in the relationship between FFM and indicators of IR in children and adolescents [38]. These controversies can be explained by many factors such as heterogeneity among the studies making them difficult to compare. For example, age of the adolescents, sample size, different ways to measure the body mass index (bioelectrical impedance, dual X-ray absorptiometry, etc.), and the way of modeling the association between body composition and IR as well as the definition of low FFMI. The latter may be caused by classification bias. For example, a Chilean adolescent’s low levels of FFMI, defined as those with values lower than the 25th percentile, was associated with IR measured by HOMA-IR [22]. In our study, we included FFMI in tertiles in the model. As a comparison we also introduced the variable of FFMI as dichotomous using 25^th^ percentile as cut-off point and the association remained not significant; similarly, we used body composition variables by their quantitative measures and the results stayed invariable. Given these results, further studies are needed to better understand the association between FFMI and HOMA-IR.

The findings of this study are of interest to public policy, as CVD and DM2 are the leading cause of death in Peru and their treatment generates high economic and social costs in the country [39]. Our results support the urgent need to promote and enhance healthy lifestyles in adolescence, including the systematic practice of physical activity as well as prevention of obesity to reduce the risk for metabolic disorders such as IR. Furthermore, early detection of IR in primary health care centers offers opportunities to start actions to tackle the onset of cardiometabolic disorders and chronic diseases in adulthood. These actions could have great impact because the early periods of life are stages when people acquire and consolidate habits and future lifestyles [40]. Considering the successful previous experience in Peru in reducing the prevalence of stunting, a major commitment and participation of stakeholders at different levels (national, regional, at community and individual level) are needed [41] to have a more effective strategy to face the NCDs related to nutrition and lifestyles. Currently, there are some initiatives to tackle obesity in Peru such as the law of promotion of healthy eating [42–45], however limited actions have been taken to improve physical activity in the adolescent population, as evidenced in the latest systematic review on interventions and policies on school environments and obesity in Latin America and the Caribbean [39]. Thus, in light of our results there is a need to strengthen the promotion of physical activity to reduce FMI and the risk for metabolic disorders in the young population in Peru.

In conclusion, in this sample of Peruvian adolescents we found that physical inactivity and high fat mass index were independently associated with increased risk for IR. Strengthened public policies to detect IR early, considering the specific metabolic characteristics or the population and implementation of actions to improve physical activity and reduction of FMI could improve the effectiveness of interventions to prevent NCDs early in life.

## References

[1] Kac G, Pérez-Escamilla R. Nutrition transition and obesity prevention through the life-course. Int J Obes Suppl. 2013;3(Suppl 1):S6–8. doi: 10.1038/ijosup.2013.3 27152157 PMC4850564

[2] Mattsson N, Rönnemaa T, Juonala M, Viikari JSA, Raitakari OT. Childhood predictors of the metabolic syndrome in adulthood. Ann Med. 2008;40(7):542–52. 18728920 10.1080/07853890802307709

[3] Corvalán C, Garmendia ML, Jones-Smith J, Lutter CK, Miranda JJ, Pedraza LS, et al. Nutrition status of children in Latin America. Obes Rev. 2017;18 Suppl 2(Suppl Suppl 2):7–18. doi: 10.1111/obr.12571 28741907 PMC5601284

[4] Lobstein T, Jackson-Leach R, Powis J, Brinsden H, Gray M. World Obesity Atlas 2023. London: World Obesity Federation. 2023. https://data.worldobesity.org/publications/?cat=19

[5] Noubiap JJ, Nansseu JR, Lontchi-Yimagou E, Nkeck JR, Nyaga UF, Ngouo AT, et al. Global, regional, and country estimates of metabolic syndrome burden in children and adolescents in 2020: a systematic review and modelling analysis. Lancet Child Adolesc Health. 2022;6(3):158–70. doi: 10.1016/S2352-4642(21)00374-6 35051409

[6] Alberti KGM, Zimmet P, Shaw J. The metabolic syndrome—a new worldwide definition. The Lancet. 2005;366(9491):1059–62. 10.1016/S0140-6736(05)67402-8 16182882

[7] Sowers JR, Standley PR, Ram JL, Jacober S, Simpson L, Rose K. Hyperinsulinemia, insulin resistance, and hyperglycemia: contributing factors in the pathogenesis of hypertension and atherosclerosis. American Journal of Hypertension. 1993;6(7_Pt_2):260S–270S. doi: 10.1093/ajh/6.7.260S 8398010

[8] Gallagher EJ, LeRoith D, Karnieli E. The metabolic syndrome--from insulin resistance to obesity and diabetes. Endocrinol Metab Clin North Am. 2008;37(3):559–79, vii. doi: 10.1016/j.ecl.2008.05.002 18775352

[9] Ormazabal V, Nair S, Elfeky O, Aguayo C, Salomon C, Zuñiga FA. Association between insulin resistance and the development of cardiovascular disease. Cardiovasc Diabetol. 2018;17(1):122. doi: 10.1186/s12933-018-0762-4 30170598 PMC6119242

[10] Zelada H, Carnero AM, Miranda-Hurtado C, Condezo-Aliaga D, Loza-Munarriz C, Aro-Guardia P, et al. Beta-cell function and insulin resistance among Peruvian adolescents with type 2 diabetes. J Clin Transl Endocrinol. 2016;5:15–20. doi: 10.1016/j.jcte.2016.05.003 29067230 PMC5644437

[11] Pajuelo J, Pando R, Leyva M, Hernández K, Infantes R. Resistencia a la insulina en adolescentes con sobrepeso y obesidad. An Fac Med. 2006;67(1):23–9.

[12] Rudvik A, Månsson M. Evaluation of surrogate measures of insulin sensitivity - correlation with gold standard is not enough. BMC Med Res Methodol. 2018;18(1):64. doi: 10.1186/s12874-018-0521-y 29940866 PMC6019831

[13] Matthews DR, Hosker JP, Rudenski AS, Naylor BA, Treacher DF, Turner RC. Homeostasis model assessment: insulin resistance and beta-cell function from fasting plasma glucose and insulin concentrations in man. Diabetologia. 1985;28(7):412–9. doi: 10.1007/BF00280883 3899825

[14] Zavaleta N, Kvistgaard AS, Graverholt G, Respicio G, Guija H, Valencia N, et al. Efficacy of an MFGM-enriched complementary food in diarrhea, anemia, and micronutrient status in infants. J Pediatr Gastroenterol Nutr. 2011;53(5):561–8. doi: 10.1097/MPG.0b013e318225cdaf 21637131

[15] Lazaro-Serrano ML, Dominguez-Curi CH. Guías alimentarias para la población peruana. 1ra ed. ed. Lima: INS. 2019.

[16] Kowalski KC, Crocker PRE, Donen RM. The Physical Activity Questionnaire for Older Children (PAQ-C) and Adolescents (PAQ-A) Manual. Saskatoon, SK: University of Saskatchewan. 2004.

[17] Bervoets L, Van Noten C, Van Roosbroeck S, Hansen D, Van Hoorenbeeck K, Verheyen E, et al. Reliability and Validity of the Dutch Physical Activity Questionnaires for Children (PAQ-C) and Adolescents (PAQ-A). Arch Public Health. 2014;72(1):47. doi: 10.1186/2049-3258-72-47 25671114 PMC4323128

[18] Montoya-Trujillano AA, Pinto-Rebatta DA, Taza-Mendoza AEF, Meléndez-Olivari EC, Alfaro-Fernández PR. Nivel de actividad física según el cuestionario PAQ-A en escolares de secundaria en dos colegios de San Martín de Porres – Lima. Rev Hered Rehab. 2016;1:21–31.

[19] Lohman TG, Roche AF, Martorell R. Anthropometric Standardization Reference Manual. Champaign, Illinois: Human Kinetics Books. 1992.

[20] Alberti SG, Zimmet P. The IDF Consensus definition of the Metabolic Syndrome in Children and Adolescents. International Diabetes Federation. 2007.

[21] Triglyceride glucose-body mass index is a simple and clinically useful surrogate marker for insulin resistance in nondiabetic individuals. Er L-K, Wu S, Chou H-H, Hsu L-A, Teng M-S, Sun Y-C, et al. Triglyceride Glucose-Body Mass Index Is a Simple and Clinically Useful Surrogate Marker for Insulin Resistance in Nondiabetic Individuals. PLoS One. 2016;11(3):e0149731. doi: 10.1371/journal.pone.0149731 26930652 PMC4773118

[22] Jiménez-Cruz A, Velasco-Martínez RM, Bacardí-Gascón M, Higuera Domínguez F, Domínguez De La Piedra E. HOMA-IR, síndrome metabólico y hábitos dietéticos en adolescentes de Chiapas, México. Rev Biomed. 2009;20(2). 19593490

[23] Burrows R, Correa-Burrows P, Reyes M, Blanco E, Albala C, Gahagan S. Healthy Chilean Adolescents with HOMA-IR ≥ 2.6 Have Increased Cardiometabolic Risk: Association with Genetic, Biological, and Environmental Factors. J Diabetes Res. 2015;2015:783296. doi: 10.1155/2015/783296 26273675 PMC4530255

[24] Chissini R de BC, Kuschnir MC, de Oliveira CL, Giannini DT, Santos B. Cutoff values for HOMA-IR associated with metabolic syndrome in the Study of Cardiovascular Risk in Adolescents (ERICA Study). Nutrition. 2020;71:110608. doi: 10.1016/j.nut.2019.110608 31783261

[25] Santos Lozano E. Resistencia a insulina: revisión de literatura. Revista Médica Hondureña. 2022;90(1):63–70. doi: 10.5377/rmh.v90i1.13824

[26] Andrade MIS de, Oliveira JS, Leal VS, Lima NM da S, Costa EC, Aquino NB de, et al. Identification of cutoff points for Homeostatic Model Assessment for Insulin Resistance index in adolescents: systematic review. Rev Paul Pediatr. 2016;34(2):234–42. doi: 10.1016/j.rpped.2015.08.006 26559605 PMC4917276

[27] Widjaja NA, Irawan R, Hanindita MH, Ugrasena IDG, Handajani R. METS-IR vs. HOMA-AD and Metabolic Syndrome in Obese Adolescents. J Med Invest. 2023;70(1.2):7–16. 37164746 10.2152/jmi.70.7

[28] Lee J, Kim M-H, Jang J-Y, Oh C-M. Assessment HOMA as a predictor for new onset diabetes mellitus and diabetic complications in non-diabetic adults: a KoGES prospective cohort study. Clin Diabetes Endocrinol. 2023;9(1):7. doi: 10.1186/s40842-023-00156-3 37974292 PMC10652621

[29] Cândido APC, Geloneze B, Calixto A, Vasques ACJ, Freitas RN, Freitas SN, et al. Adiponectin, HOMA-Adiponectin, HOMA-IR in Children and Adolescents: Ouro Preto Study. Indian J Pediatr. 2021;88(4):336–44. doi: 10.1007/s12098-020-03444-3 32945992

[30] Rocca-Nación J, Calderon M. Cardiovascular risk, fatty liver disease, glucose and insulin curve among prediabetes phenotypes in Peruvian population. Am J Med Open. 2022;7:100007. doi: 10.1016/j.ajmo.2022.100007 39035828 PMC11256264

[31] Guinhouya BC, Samouda H, Zitouni D, Vilhelm C, Hubert H. Evidence of the influence of physical activity on the metabolic syndrome and/or on insulin resistance in pediatric populations: a systematic review. Int J Pediatr Obes. 2011;6(5–6):361–88. doi: 10.3109/17477166.2011.605896 21851163

[32] Fraemke A, Ferrari N, Friesen D, Haas F, Klaudius M, Mahabir E. HOMA Index, Vitamin D Levels, Body Composition and Cardiorespiratory Fitness in Juvenile Obesity: Data from the CHILT III Programme, Cologne. Int J Environ Res Public Health. 2022;19(4). 10.3390/ijerph19042442 PMC8872273 35206632

[33] Sedlmeier AM, Baumeister SE, Weber A, Fischer B, Thorand B, Ittermann T, et al. Relation of body fat mass and fat-free mass to total mortality: results from 7 prospective cohort studies. Am J Clin Nutr. 2021;113(3):639–46. doi: 10.1093/ajcn/nqaa339 33437985

[34] Liu P, Ma F, Lou H, Liu Y. The utility of fat mass index vs. body mass index and percentage of body fat in the screening of metabolic syndrome. BMC Public Health. 2013;13:629. doi: 10.1186/1471-2458-13-629 23819808 PMC3703297

[35] Warolin J, Coenen KR, Kantor JL, Whitaker LE, Wang L, Acra SA, et al. The relationship of oxidative stress, adiposity and metabolic risk factors in healthy Black and White American youth. Pediatr Obes. 2014;9(1):43–52. doi: 10.1111/j.2047-6310.2012.00135.x 23296459 PMC3775931

[36] Benjamin EJ, Virani SS, Callaway CW, Chamberlain AM, Chang AR, Cheng S. Heart Disease and Stroke Statistics-2018 Update: A Report From the American Heart Association. Circulation. 2018;137. 10.1161/CIR.0000000000000558 29386200

[37] Świątkiewicz I, Wróblewski M, Nuszkiewicz J, Sutkowy P, Wróblewska J, Woźniak A. The Role of Oxidative Stress Enhanced by Adiposity in Cardiometabolic Diseases. Int J Mol Sci. 2023;24(7):6382. doi: 10.3390/ijms24076382 37047352 PMC10094567

[38] Wu L, Chen F, Liu J, Hou D, Li T, Chen Y, et al. The Relationship Between Fat-Free Mass and Glucose Metabolism in Children and Adolescents: A Systematic Review and Meta-Analysis. Front Pediatr. 2022;10:864904. doi: 10.3389/fped.2022.864904 35558370 PMC9087035

[39] Bloom DE, Chen S, McGovern ME. The economic burden of noncommunicable diseases and mental health conditions: results for Costa Rica, Jamaica, and Peru. Rev Panam Salud Publica. 2018;42:e18. doi: 10.26633/RPSP.2018.18 31093047 PMC6386108

[40] Krauskopf D. El desarrollo psicológico en la adolescencia: las transformaciones en una época de cambios. Adolesc Salud. 1999;1(2).

[41] Marini AL, Rokx C. Dando la talla: El éxito del Perú en la lucha contra la desnutrición crónica. Washington DC: Grupo Banco Mundial.

[42] Vega-Salas MJ, Murray C, Nunes R, Hidalgo-Arestegui A, Curi-Quinto K, Penny ME, et al. School environments and obesity: a systematic review of interventions and policies among school-age students in Latin America and the Caribbean. Int J Obes (Lond). 2023;47(1):5–16. doi: 10.1038/s41366-022-01226-9 36216909 PMC9549440

[43] Congreso de la República del Perú. Normas legales 2007. https://www2.congreso.gob.pe/sicr/cendocbib/con4_uibd.nsf/5289E04A2A160ABD052581A10070E6CE/%24FILE/2_decreto_supre_017_de_alimentacion.pdf Accessed 2023 October 1.

[44] Organización Panamericana de la Salud. Ley de promoción de la alimentación saludable para niñas, niños y adolescentes. Experiencia en Perú. Lima: OPS. 2021.

[45] Anastasiou CA, Fappa E, Zachari K, Mavrogianni C, Van Stappen V, Kivelä J, et al. Development and reliability of questionnaires for the assessment of diet and physical activity behaviors in a multi-country sample in Europe the Feel4Diabetes Study. BMC Endocr Disord. 2020;20(Suppl 1):135. doi: 10.1186/s12902-019-0469-x 32164677 PMC7066729

