# Supplementary material for: Identification of a HOMA-IR cut-off point for cardiometabolic risk and modifiable risk factors in peruvian adolescents
Source: PLoS One. 2026 Jul 10;21(7):e0351139. doi: 10.1371/journal.pone.0351139 (PMC13354073; doi:10.1371/journal.pone.0351139)
Supplement: Attachment — Submitted filename: Reviewer Comments on PONE-D-25-02583_R2.pdf [file pone.0351139.s005.pdf]

Dear Editor,

The authors have made a commendable effort to address the concerns raised during the previous round of review. The inclusion of new literature, the repositioning of figures, and the clarification of methodological details have noticeably strengthened the manuscript.

Before this manuscript can be accepted for publication, the authors must address the following minor but critical corrections:

**Abstract Sample Size Inconsistency:** In response to Reviewer #2 regarding sample size confusion, the authors clarified in the main text that the total sample is 371, with a sub-sample of 349 used specifically for FFM/FFMI analyses. Unfortunately, the authors neglected to update the Abstract, which still states: *"A sample of 349 adolescents..."*. The Abstract must be updated to reflect the full cohort of 371.

**Abstract Diagnostic Overstatement:** The authors agreed to remove the subjective descriptor "high" when referring to sensitivity and specificity, modifying the Discussion accordingly. However, this overstatement was left intact in the Abstract, which still reads: *"...with high sensitivity (72.4%) and specificity (75.4%)."*. This must be amended to match the neutral tone of the main text.

**Scientific Typographical Error in Table 1:** While the authors successfully corrected the HOMA-IR formula units in the Methods section (Lines 170-172) to µU/mL and mmol/L, the footnote for Table 1 contains a major scientific typo. It defines the calculation using fasting insulin in **µU/L** and fasting glucose in **nmol/L**. These units are incorrect for a divisor of 22.5 and must be corrected to match the main text.

**Recommendation: Minor Revisions.**

The core science, data, and conclusions of the study are sound. Once the authors align the Abstract and Table 1 footnotes with the corrections, they have already established in the main text, the manuscript will be highly suitable for publication and will make a valuable contribution to the understanding of adolescent metabolic risk in Latin America.

Sincerely,
