# Supplementary material for: Identification of a HOMA-IR cut-off point for cardiometabolic risk and modifiable risk factors in peruvian adolescents
Source: PLoS One. 2026 Jul 10;21(7):e0351139. doi: 10.1371/journal.pone.0351139 (PMC13354073; doi:10.1371/journal.pone.0351139)
Supplement: Attachment — Submitted filename: Reviewer Comments_PONE-D-25-02583.docx [file pone.0351139.s001.docx]

Dear Dr. Neftali Eduardo Antonio-Villa,

This study addresses an important public health issue by establishing a HOMA-IR cut-off point for identifying insulin resistance (IR) and associated cardiometabolic risks in Peruvian adolescents, alongside identifying modifiable risk factors such as physical inactivity and fat mass index (FMI). The research is timely and relevant, given the rising prevalence of obesity and non-communicable diseases (NCDs) in adolescent populations globally, particularly in middle-income settings like Peru.

The manuscript presents a well-conducted secondary analysis of longitudinal data, employing robust statistical methods such as ROC curve analysis and multivariate logistic regression. The findings are generally consistent and scientifically sound, with a HOMA-IR cut-off of 3.9 identified for metabolic syndrome (MS) prediction, supported by reasonable sensitivity (72.4%) and specificity (75.4%). The discussion appropriately contextualizes these results within existing literature and highlights actionable implications for public health policy.

However, I have identified several areas requiring attention to enhance scientific accuracy, consistency, and readability. These include minor discrepancies in reported sample sizes (e.g., 349 vs. 371 participants), incomplete sentences in the abstract and results, and occasional grammatical errors that could affect clarity. Additionally, some methodological details (e.g., justification for tertile classification of FMI/FFMI) and discussion points (e.g., exploration of sex differences) could be expanded for clarity and depth. I have provided a detailed review below, addressing these issues alongside PLOS ONE’s submission criteria. My detailed comments include concerns regarding the statistical analysis, the clarity of methodology, and the consistency of results and discussion. Additionally, some sections require grammatical refinement to enhance readability.

After a thorough review adhering to PLOS ONE criteria, I recommend **major revisions** to address the identified scientific inconsistencies, clarify methodological choices, correct grammatical errors, and overall impact. With these adjustments, the manuscript will meet *PLOS ONE*’s standards for publication and contribute valuable insights into the field of adolescent metabolic health.

**PLOS ONE Submission Criteria Assessment**

1. **Originality**: The study is original, providing the first reported HOMA-IR cut-off for Peruvian adolescents, a population underrepresented in metabolic research. It builds on prior global studies while offering country-specific insights.
2. **Scientific Accuracy**: The methodology is convincing, utilizing ROC curve analysis and logistic regression. However, minor inconsistencies (e.g., sample size reporting) and incomplete reporting of ethical consent documentation details require clarification.
3. **Clarity and Conciseness**: The manuscript is generally clear but contains grammatical errors and incomplete sentences (e.g., abstract results section). Streamlining and proofreading will enhance readability.
4. **Ethical Standards**: An ethics statement is provided, noting approval by the Nutritional Research Institute and informed consent/assent. However, specifics on how consent was documented are vague and need elaboration.
5. **Financial Disclosure**: Funding from Universidad Peruana de Ciencias Aplicadas (UPC-EXPOST-2024-1) and original study funders (Mead Johnson & Arla Foods) is disclosed, with no role in study design or analysis, meeting transparency requirements.

**Comments**

**Abstract (Pages 1, 9-10)**

- **Abstract (Lines 27-52, Page 10)**:
  - **Line 28**: "Currently, there is a need..." – Grammatically correct but could be more concise: "Promoting healthy lifestyles in adolescence is critical..."
  - **Line 33**: "A sample of 349 adolescents" – Consistent with methods (Line 115, Page 13), but Table 1 (Page 32) reports n=371. This discrepancy needs resolution.
  - **Line 39**: "The HOMA-IR was 3.29 (SD 1.71)" – Should clarify this is the mean (as in Results, Line 211, Page 17). Suggest: "Mean HOMA-IR was 3.29 (SD 1.71)."
  - **Line 43**: "No association was found with diet, excess weight at infancy and." – Incomplete sentence. Suggest: "No association was found with diet, excess weight at infancy, or family history of chronic diseases."
  - **Line 45**: "A cut-offs point" – Grammatical error; should be "A cut-off point."

**Introduction (Pages 11-12)**

- **Line 53-55 (Page 11)**: "Early exposure to negative changes in lifestyle such as dietary patterns and physical activity have increased the risk for obesity and cardiometabolic disorders earlier in life [1-3]."
  - **Comment**: This opening effectively defines the problem by linking lifestyle changes to obesity and cardiometabolic risks. However, "negative changes" is vague—specifying "unhealthy diets" or "sedentary behavior" would clarify the scope. The citations support the claim well.
- **Line 54**: "Early exposure to negative changes in lifestyle" – Slightly awkward phrasing. Suggest: "Early adoption of unhealthy lifestyles."
- **Line 56**: "10 to $17 %$ in mails" – Typo; should be "males."
- **Line 56-58**: "According to the World Atlas of Obesity 2023, children and adolescents are the most vulnerable population because the prevalence of obesity in these groups is likely to increase from 2020 to 2030 by 10 to 17% in mails and from 8% to 14% in females [4]."
  - **Comment**: Provides concrete evidence of the problem’s scale, though "mails" is a typo (should be "males"). Including a Peruvian statistic here (if available) would localize the issue further.
- **Line 58-61**: "Along with obesity, there is an early onset of cardiometabolic disorders such as metabolic syndrome (MS)... prevalence of MS was 5.5% (4.1-8.4) in high-income countries... 7.0% (2.4-15.7) in low-income countries [5]."
  - **Comment**: Effectively broadens the problem to MS, with global prevalence data. However, no Peruvian-specific MS prevalence is mentioned, which could strengthen the rationale for a local study.
- **Line 62-68**: "MS is a cluster of cardiometabolic disorders that includes abdominal obesity, glucose intolerance, hypertension, and dyslipidemia [6]... increases the risk for chronic noncommunicable diseases (NCDs) such as type 2 diabetes and cardiovascular disease in adulthood [7,8]."
  - **Comment**: Clearly defines MS and ties IR to long-term health consequences, supported by citations. This sets up the scientific basis well but could briefly note modifiable factors (e.g., diet, activity) as intervention targets.
- **Line 62**: "Insulin resistance has been considered an underlying factor" – Accurate, but could cite a foundational reference (e.g., Reaven, 1988) for context.
- **Line 62**: "...includes abdominal obesity, glucose intolerance, hypertension, and dyslipidemia [6)." – Typo; change bracket to "]".
- **Line 71-73**: "Homeostasis model assessment-estimated insulin resistance (HOMA-IR) is the most common method used to measure IR [9,10]... a more accessible and non-invasive method compared to the gold standard of hyperinsulinemic euglycemic clamp [11,12]."
  - **Comment**: Introduces HOMA-IR effectively, highlighting its practicality. This supports the study’s methodology but could mention its adaptability to population-specific cut-offs here.
- **Line 75-77**: "HOMA-IR is affected by different factors... ethnicity, age, sex, and metabolic conditions... necessary to identify country specific HOMA-IR cut-off points... no studies in Peruvian adolescents that have suggested specific cut-off points for HOMA-IR classification."
  - **Comment**: Justifies the need for a Peruvian-specific cut-off, defining a research gap. The claim of no prior studies is bold—consider softening to "few studies" unless a systematic review confirms this.
- **Line 77**: "To the best of our knowledge" – Appropriate, but no systematic literature review is cited to support this claim. Consider referencing a scoping review if available.
- **Line 78-80**: "Therefore, this study aimed to determine the distribution of HOMA-IR values and identify a cut-off value associated with MS, as well as identifying the modifiable risk factors of IR in a longitudinal study of Peruvian adolescents."
  - **Comment**: The aim is clear and aligns with the problem (IR/MS detection) and gap (country-specific data). It hints at solutions (identifying modifiable factors) but could explicitly connect these to prevention strategies (e.g., "to inform targeted interventions").
- **Consistency**: The introduction aligns with the study’s aims and sets up the need for a country-specific HOMA-IR cut-off.

**Suggestions for Improvement**

1. **Comprehensiveness**: Add a sentence on modifiable risk factors (e.g., "Factors like physical inactivity and excess adiposity are key drivers amenable to intervention") to bridge the problem and solution.
2. **Local Context**: Include Peruvian obesity/MS data (if available) to localize the problem beyond global trends.
3. **Solution Pathway**: Strengthen the link to interventions by noting how identifying cut-offs and risk factors can guide public health actions (e.g., screening or lifestyle programs).

**Materials and Methods (Pages 13-16)**

- **Study Design and Population (Lines 105-118, Page 13)**:
  - **Line 107**: "...349 participants that have complete data..." – Change "that have" to "who have" for grammatical correctness.
  - **Line 115**: "From a total of 394 adolescents, we analyzed data of 349 participants" – Conflicts with Table 1 (n=371). Clarify the correct sample size and exclusion criteria.
  - **Line 117**: Ethics approval is noted, but consent documentation details are incomplete (repeated in Ethics Statement, Page 4). Specify how consent was recorded (e.g., signed forms).
- **Adolescence Nutritional Status (Lines 119-126)**:
  - **Line 122**: "SECA scale" and "Holstein stadiometer" – Brand names are fine, but ensure consistency in capitalization (e.g., "Seca" elsewhere).
  - **Line 125**: "Values <2SD were underweight" – Should be "<-2 SD" for consistency with WHO standards.
- **Adolescent Cardiometabolic Risk Factors (Lines 128-135)**:
  - **Line 131**: "Fasting serum total glucose" – "Total glucose" is unusual; typically "fasting glucose." Verify terminology.
  - **Line 131**: "...was measured in venous blood sample..." – Change to "venous blood samples".
  - **Line 133**: "Systolic and diastolic blood pressures" – Add units (mmHg) for clarity in the methods.
- **Adolescent Body Composition (Lines 137-141)**:
  - **Line 139**: "FMI and FFMI were estimated" – Justify why tertiles were used instead of continuous variables or established cut-offs.
- **Diet and Physical Activity (Lines 143-157)**:
  - **Line 148**: "We defined the diet as relatively healthy or unhealthy whether a person fulfilled..." – Awkward phrasing. Suggest: "Diet was classified as healthy or unhealthy based on meeting recommended intake for at least four food groups."
  - **Line 155**: "The final score was obtained by the arithmetic average" – Clarify if this is a mean score (1-5 scale) and define "low physical activity" threshold (e.g., <3, as in Table 4).
- **Nutritional Status at Infancy (Lines 159-166)**:
  - **Line 163**: "Childhood overweight and obesity was diagnosed" – Should be "were diagnosed."
- **Definition of Metabolic Syndrome (Lines 168-172)**:
  - **Line 170**: Criteria are correctly cited (IDF-2007), but specify if adapted for adolescents (e.g., WC percentiles).
- **Definition of Insulin Resistance (Lines 176-184)**:
  - **Line 177**: HOMA-IR formula is correct. ROC analysis description is sound but could note software used (assumed Stata from Line 204).
- **Statistical Analysis (Lines 195-204)**:
  - **Line 199**: "Bivariate binomial logistic regression" – Redundant; "bivariate logistic regression" suffices.
  - **Line 201**: "Model 3" and "Model 4" – Should be "Model 1" and "Model 2" to match Table 4 (Page 35).

**Results (Pages 17-18)**

**Overall Assessment**

The Results section presents key findings logically, starting with sample characteristics, HOMA-IR distribution, cut-off determination, and risk factor associations. Data generally align with tables, but minor inconsistencies (e.g., sample size, blood pressure values) and incomplete reporting (e.g., sex differences) require attention to ensure no contradictions with the Discussion.

- **Line 211 (Page 17)**: "Mean age of the adolescents was 14.5 (0.1 SD) years, and $48.1% of the population was female. Mean HOMA-IR was 3.3 (95% CI 3.1; 3.5%)."
  - **Comment**: Matches Methods (Line 115, n=349) but not Table 1 (n=371). CI should be "3.1-3.5" (no %). sample size needs clarification.
- **Line 212**: "Prevalence of obesity and overweight" – Matches Table 2 but clarify if z-BMI-based.
- **Line 214-215**: "Table 1 shows the distribution of the HOMA-IR percentiles by sex, body mass index and MS."
  - **Comment**: Table 1 reports n=371, mean HOMA-IR 3.29 (SD 1.71), slightly differing from 3.3 (CI 3.1-3.5). Minor rounding discrepancy; clarify true value.
- **Line 216-217**: "The mean HOMA-IR was significantly higher in overweight and obese adolescents compared with those with healthy weight $(3.42; 5.17 vs 2.75)$, and those with MS compared with those without MS (5.31 vs 3.11)."
  - **Comment**: Matches Table 1 means. "No significant difference was found by sex" aligns with Table 1 (3.30 vs. 3.27), but Discussion doesn’t explore this despite Table 4’s sex effect.
- **Line 218-219**: "Table 1 presents the optimal cutoff points for HOMA-IR to predict MS in males and females. A HOMA-IR with a cutoff of 3.9 showed a sensitivity of $72.4% and a specificity of $75.4 %$; with an area under the curve ROC of 0.79 (IC 0.69 0.88)."
  - **Comment**: Table 1 doesn’t stratify cut-offs by sex—text misstates this. AUC CI should be "0.69-0.88."
- **Line 218**: "A HOMA-IR with a cutoff of 3.9" – Remove "with" for conciseness: "A HOMA-IR cutoff of 3.9."
- **Line 220-222**: "Table 2 displays the anthropometric and cardiometabolic adolescent profile by IR status... significantly higher values of z-score for BMI/age, body composition indicators (FFMI and FMI) and cardiometabolic indicators..."
  - **Comment**: Matches Table 2, but systolic BP (14.9 mmHg, Table 2) is implausible (likely 114.9 mmHg). Correct this to avoid contradiction.
- **Line 222**: "...waist circumference, triglycerides, glucose, blood pressure, and insulin; while the HDL was significantly lower." – Change semicolon to a period for better sentence structure.
- **Line 226**: "At least six out of ten" – Could be simplified: "62% of adolescents."
- **Line 226-230**: "The proportion of cardiometabolic risk factors by IR is presented in Figure 2... at least six out of ten adolescents had at least one cardiometabolic risk factor (62%)... low levels of HDL (62.3% and $39.6 %$, respectively)."
  - **Comment**: Figure 2 aligns (62% total for low HDL), but Table 2 mean HDL (46.7 mg/dl) doesn’t directly confirm prevalence—clarify calculation method. "39.6%" seems incorrect (should be non-IR prevalence); verify.
- **Line 233-234**: "The prevalence of all the cardiometabolic risk factors was significantly higher in adolescents with IR compared with those without IR..."
  - **Comment**: Matches Figure 2 and Table 2 (p=0.001 for diastolic BP), but systolic BP typo affects interpretation. Adjust accordingly.
- **Line 235-238**: "Table 3 presents the results of the bivariate analysis... Low levels of physical activity, overweight and obesity in adolescence, FMI, FFMI and presence of excess weight were significantly associated with the presence of MS."
  - **Comment**: Text says "MS," but context implies "IR" (Table 3 is IR-based). Correct this. Data match Table 3.
- **Line 240-244**: "In the adjusted analysis using multiple logistic regression models (Table 4), low physical activity and high FMI were independently associated to IR... None of this variable has been associated with the IR."
  - **Comment**: Matches Table 4 (OR 2.08 for low PA, 16.03 for high FMI). "Female" OR 2.78 contradicts Table 3 (OR 1.10)—explain adjustment effect.
- **Line 244**: "None of this variable has been associated" – Grammatical error; should be "None of these variables were associated."

**Discussion (Pages 19-21)**

**Overall Assessment**

The Discussion contextualizes the HOMA-IR cut-off (3.9) and risk factors (physical inactivity, FMI) within existing literature, offering policy implications. Most data align with Results, but minor mismatches (e.g., prevalence rates) and unexplored findings (e.g., sex effect) need clarification.

- **Line 261**: "In this study we identified a value of 3.90 as specific cut-off point for HOMA-IR..." – Clarify why 3.90 was chosen over other nearby values.
- **Line 262**: "A value of 3.90" – Use consistent decimals (3.9 elsewhere).
- **Line 264-266**: "According to this cut-off point, 3 out of 10 adolescents had IR... low HDL (62%), abdominal obesity (35%), hypertriglyceridemia (23%), fasting hyperglycemia (8.5%), and MS (20%)."
  - **Comment**: IR prevalence (28.6%, Abstract Line 41) aligns with "3 out of 10." HDL 62% matches Figure 2, but abdominal obesity (35%) and others differ slightly from Table 2/Figure 2—clarify source. MS 20% contradicts Results (7.8%, Line 214)—major error.
- **Line 276**: "HOMA-IR was associated with metabolic risk" – Supported by results, but no sex differences were discussed despite Table 1 stratification. Consider addressing this.
- **Line 276**: "...HOMA-IR was associated with metabolic risk, and it was the most suitable methods..." – Change "methods" to "method".
- **Line 293**: "Showing greater importance of the role" – Awkward; suggest: "Indicating the greater role of adipose tissue in IR."
- **Line 295**: "...classification bias. For example, in Chilean adolescent’s low levels..." – Apostrophe misuse; change to "adolescents".
- **Line 304**: "Further studies are needed" – Valid, but specify what gaps (e.g., FFMI measurement standardization).

**Conclusion**:

- **Line 320**: "...considering the specific metabolic characteristics or the population..." – Change "or" to "of".

**Tables and Figures**

- **Figure 2**: Clarify the meaning of error bars in the legend.
- **Table 1 (Page 32)**: n=371 vs. 349 elsewhere. Resolve this inconsistency.
- **Table 2 (Page 32-33)**: Systolic BP mean (14.9 mmHg) is implausible; likely a typo (e.g., 114.9 mmHg).
- **Table 4 (Page 35)**: "Female OR 2.78" – Conflicts with bivariate analysis (OR 1.10, Table 3). Verify adjustment effects or correct.

**References (Pages 23-27)**

- Formatting is mostly consistent, but some URLs (e.g., Ref 4) could be shortened with DOIs if available.

**Summary of Key Issues**

1. **Scientific Inconsistency**: Sample size discrepancy (349 vs. 371), systolic BP error in Table 2, and unexpected sex effect in Table 4 need resolution.
2. **Methodological Clarity**: Justify tertile use for FMI/FFMI and clarify consent documentation.
3. **Grammatical Errors**: Minor but frequent (e.g., "cut-offs point," incomplete sentences).
4. **Discussion Depth**: Expand on sex differences and FFMI findings.
