# Supplementary material for: Identification of a HOMA-IR cut-off point for cardiometabolic risk and modifiable risk factors in peruvian adolescents
Source: PLoS One. 2026 Jul 10;21(7):e0351139. doi: 10.1371/journal.pone.0351139 (PMC13354073; doi:10.1371/journal.pone.0351139)
Supplement: Attachment — Submitted filename: RESPONSE LETTER 26 01 26.docx [file pone.0351139.s004.docx]

**RESPONSE LETTER**

Lima, 26 January 2026

**PONE-D-25-02583R1
IDENTIFICATION OF A HOMA-IR CUT-OFF POINT FOR CARDIOMETABOLIC RISK AND MODIFIABLE RISK FACTORS IN PERUVIAN ADOLESCENTS

Editor Comments**

1. Please remove your figures/ from within your manuscript file, leaving only the individual TIFF/EPS image files.  These will be automatically included in the reviewer’s PDF

We have removed the figures from the main manuscript and will attach them as separate files in TIFF format. The figure legends have been placed on the last pages of the manuscript.
