# Supplementary material for: Identification of a HOMA-IR cut-off point for cardiometabolic risk and modifiable risk factors in peruvian adolescents
Source: PLoS One. 2026 Jul 10;21(7):e0351139. doi: 10.1371/journal.pone.0351139 (PMC13354073; doi:10.1371/journal.pone.0351139)
Supplement: Attachment — Submitted filename: Reviewer Comments on PONE-D-25-02583_R1.docx [file pone.0351139.s003.docx]

Dear Editor,

I have carefully reviewed the revised version of manuscript PONE-D-25-02583R1 entitled *“Identification of a HOMA-IR cut-off point for cardiometabolic risk and modifiable risk factors in Peruvian adolescents.”*

The authors have made substantial efforts to address reviewer comments. The manuscript has improved in structure, clarity, and contextualization. The study addresses an important public health issue and provides regionally relevant data on insulin resistance and metabolic risk in adolescents.

However, several critical issues remain unresolved in the revised manuscript:

1. Persistent inconsistencies in reported sample size (349 vs 371 participants across sections and tables).
2. Implausible systolic blood pressure values are still present in Table 2.
3. Ethical section contains placeholder text that has not been properly revised.
4. Ambiguity in the HOMA-IR calculation formula and unit reporting.
5. Overstatement of diagnostic performance (sensitivity/specificity described as “high”).
6. Minor but recurrent grammatical and reporting inconsistencies.

While these issues appear correctable, they are substantial and affect the scientific reliability and reporting integrity of the manuscript.

Therefore, I recommend **Major Revision** before the manuscript can be considered for acceptance.

Once numerical inconsistencies, methodological clarifications, and reporting errors are fully corrected, the manuscript has the potential to make a meaningful contribution to the literature on adolescent metabolic risk in Latin America.

Sincerely,
