# Supplementary material for: Identification of a HOMA-IR cut-off point for cardiometabolic risk and modifiable risk factors in peruvian adolescents
Source: PLoS One. 2026 Jul 10;21(7):e0351139. doi: 10.1371/journal.pone.0351139 (PMC13354073; doi:10.1371/journal.pone.0351139)
Supplement: Attachment — Submitted filename: Response Letter 07 04 2026.docx [file pone.0351139.s006.docx]

PONE-D-25-02583R1

IDENTIFICATION OF A HOMA-IR CUT-OFF POINT FOR CARDIOMETABOLIC RISK AND MODIFIABLE RISK FACTORS IN PERUVIAN ADOLESCENTS

PLOS One

Dear Dr. Curi-Quinto,

Thank you for submitting your manuscript to PLOS ONE. After careful consideration, we feel that it has merit but does not fully meet PLOS ONE’s publication criteria as it currently stands. Therefore, we invite you to submit a revised version of the manuscript that addresses the points raised during the review process.

Please submit your revised manuscript by Apr 27 2026 11:59PM. If you will need more time than this to complete your revisions, please reply to this message or contact the journal office at [plosone@plos.org](mailto:plosone@plos.org). When you're ready to submit your revision, log on to <https://www.editorialmanager.com/pone/> and select the 'Submissions Needing Revision' folder to locate your manuscript file.

Please include the following items when submitting your revised manuscript:

- A letter that responds to each point raised by the academic editor and reviewer(s). You should upload this letter as a separate file labeled 'Response to Reviewers'.
- A marked-up copy of your manuscript that highlights changes made to the original version. You should upload this as a separate file labeled 'Revised Manuscript with Track Changes'.
- An unmarked version of your revised paper without tracked changes. You should upload this as a separate file labeled 'Manuscript'.

If you would like to make changes to your financial disclosure, please include your updated statement in your cover letter. Guidelines for resubmitting your figure files are available below the reviewer comments at the end of this letter.

If applicable, we recommend that you deposit your laboratory protocols in [protocols.io](http://protocols.io/) to enhance the reproducibility of your results. Protocols.io assigns your protocol its own identifier (DOI) so that it can be cited independently in the future. For instructions see: [https://journals.plos.org/plosone/s/submission-guidelines#loc-laboratory-protocols](https://track.editorialmanager.com/CL0/https:%2F%2Fjournals.plos.org%2Fplosone%2Fs%2Fsubmission-guidelines%23loc-laboratory-protocols/1/010f019ce586750d-8cc3a999-90ed-4489-86cd-6a8d2b3e3230-000000/SQ_6RLpbXIi_bwFDjDZVfpXPrfFA-m1Zj7mMN1Fd7Ko=254). Additionally, PLOS ONE offers an option for publishing peer-reviewed Lab Protocol articles, which describe protocols hosted on [protocols.io](http://protocols.io/). Read more information on sharing protocols at [https://plos.org/protocols?utm_medium=editorial-email&utm_source=authorletters&utm_campaign=protocols](https://track.editorialmanager.com/CL0/https:%2F%2Fplos.org%2Fprotocols%3Futm_medium=editorial-email%26utm_source=authorletters%26utm_campaign=protocols/1/010f019ce586750d-8cc3a999-90ed-4489-86cd-6a8d2b3e3230-000000/dC-FXOp7_kcQl2fl41lNsJa31PubrusmASAWsjfmaBM=254).

We look forward to receiving your revised manuscript.

Kind regards,

Neftali Eduardo Antonio-Villa, MD PhD
Academic Editor
PLOS One

Journal Requirements:

If the reviewer comments include a recommendation to cite specific previously published works, please review and evaluate these publications to determine whether they are relevant and should be cited. There is no requirement to cite these works unless the editor has indicated otherwise. 


[Note: HTML markup is below. Please do not edit.]

Reviewers' comments:

Reviewer's Responses to Questions

**Comments to the Author**

1. If the authors have adequately addressed your comments raised in a previous round of review and you feel that this manuscript is now acceptable for publication, you may indicate that here to bypass the “Comments to the Author” section, enter your conflict of interest statement in the “Confidential to Editor” section, and submit your "Accept" recommendation.

Reviewer #1: All comments have been addressed

Reviewer #2: All comments have been addressed

Reviewer #5: (No Response)

2. Is the manuscript technically sound, and do the data support the conclusions?

The manuscript must describe a technically sound piece of scientific research with data that supports the conclusions. Experiments must have been conducted rigorously, with appropriate controls, replication, and sample sizes. The conclusions must be drawn appropriately based on the data presented.

Reviewer #1: Yes

Reviewer #2: Partly

Reviewer #5: Yes

3. Has the statistical analysis been performed appropriately and rigorously?

Reviewer #1: Yes

Reviewer #2: No

Reviewer #5: I Don't Know

4. Have the authors made all data underlying the findings in their manuscript fully available?

The [PLOS Data policy](http://track.editorialmanager.com/CL0/http:%2F%2Fwww.plosone.org%2Fstatic%2Fpolicies.action%23sharing/1/010f019ce586750d-8cc3a999-90ed-4489-86cd-6a8d2b3e3230-000000/-c93BYoszGXD9d1ypLMYuyQ_CviEk4UpFWXQGnl3Jvw=254) requires authors to make all data underlying the findings described in their manuscript fully available without restriction, with rare exception (please refer to the Data Availability Statement in the manuscript PDF file). The data should be provided as part of the manuscript or its supporting information, or deposited to a public repository. For example, in addition to summary statistics, the data points behind means, medians and variance measures should be available. If there are restrictions on publicly sharing data—e.g. participant privacy or use of data from a third party—those must be specified.

Reviewer #1: Yes

Reviewer #2: Yes

Reviewer #5: No

5. Is the manuscript presented in an intelligible fashion and written in standard English?

PLOS ONE does not copyedit accepted manuscripts, so the language in submitted articles must be clear, correct, and unambiguous. Any typographical or grammatical errors should be corrected at revision, so please note any specific errors here.

Reviewer #1: Yes

Reviewer #2: Yes

Reviewer #5: Yes

6. Review Comments to the Author

Please use the space provided to explain your answers to the questions above. You may also include additional comments for the author, including concerns about dual publication, research ethics, or publication ethics. (Please upload your review as an attachment if it exceeds 20,000 characters)

Reviewer #1: I have no additional comments to improve the resubmission. All of my concerns were addressed by the author.

**Reviewer #2: Dear Editor,**

I have carefully reviewed the revised version of manuscript PONE-D-25-02583R1 entitled “Identification of a HOMA-IR cut-off point for cardiometabolic risk and modifiable risk factors in Peruvian adolescents.”
The authors have made substantial efforts to address reviewer comments. The manuscript has improved in structure, clarity, and contextualization. The study addresses an important public health issue and provides regionally relevant data on insulin resistance and metabolic risk in adolescents.
However, several critical issues remain unresolved in the revised manuscript:

**1. Persistent inconsistencies in reported sample size (349 vs 371 participants across sections and tables).**

Thank you for your comment.
To clarify, all our main analyses were conducted using a sample of 371 participants. However, data on Fat Free Mass and Fat Free Mass Index were available for only 349 participants. It is important to note that the subset of 349 participants was used exclusively for a regression analysis (Table 3) and not for the main analyses, such as the identification of HOMA-IR.

Therefore, to avoid confusion, we have clarified this in the *Study Design and Population* section and have corrected the remaining inconsistencies throughout the manuscript.

From a total of 394 adolescents, we analyzed data of 371 participants that have complete data from infancy and adolescence. For fat-free mass (FFM) and fat-free mass index (FFMI), data were available for 349 participants; thus, related bivariate analyses used this subsample, while others used the full sample (n = 371).

See lines: 116 - 117

**2. Implausible systolic blood pressure values are still present in Table 2.**

Thank you for your observation. We acknowledge that there was a typographical error, which has now been corrected.

See lines: 223

**3. Ethical section contains placeholder text that has not been properly revised.**

Thank you for identifying the error. We have corrected it as follows:

The ethics committees of the Instituto de Investigación Nutricional (IIN), Lima-Peru, approved this study under the number 372-2017/CIEI-IIN. This study was conducted in accordance with the ethical principles of the Declaration of Helsinki. We obtained written informed consent from all participants prior to their inclusion as well as the assent from the adolescents.

See lines: 180-183

**4. Ambiguity in the HOMA-IR calculation formula and unit reporting.**

Thank you for the clarification. We have revised the wording of this section and have also added a relevant reference.

IR was estimated using the HOMA-IR, calculated as the product of fasting insulin (µU/mL) and fasting glucose (mmol/L), divided by 22.5. This index provides an indirect measure of insulin sensitivity based on fasting metabolic parameters [21].

See lines: 170-172

**5. Overstatement of diagnostic performance (sensitivity/specificity described as “high”).**

Thank you for the clarification. In the Discussion section, we have revised this statement to a more neutral wording and removed the overstatement, as follows:

In this study we identified a value of 3.90 as specific cut-off point for HOMA-IR that has a sensitivity of 72.4% and specificity of 75.4% for predicting MS in Peruvian adolescents with an average age of 14.5 years (AUC: 0.79; 95% CI 0.69–0.88).

See lines: 246-248

**6. Minor but recurrent grammatical and reporting inconsistencies.**

The manuscript has been thoroughly reviewed, and all errors have been corrected.

While these issues appear correctable, they are substantial and affect the scientific reliability and reporting integrity of the manuscript.
Therefore, I recommend Major Revision before the manuscript can be considered for acceptance.
Once numerical inconsistencies, methodological clarifications, and reporting errors are fully corrected, the manuscript has the potential to make a meaningful contribution to the literature on adolescent metabolic risk in Latin America.
Sincerely,

**Reviewer #5:**

This article by Curi-Quinto and collaborators is entitled : « Identification of a HOMA-IR cut-OFF point for cardiometabolis risk and modifiable risk factors in Peruvian adolescents ». This is the first revised version of a manuscript firstly sent last year to pone journal.

The aim of this article is to identify cut-off values from HOMA-IR assay in a cohort of Peruvian adolescents in order to propose an anticipated diagnosis for metabolic syndrome.

The Table 1 represents the percentile distribution of the HOMA-IR in the cohort studied and with criteria stratifications. Means from Z-score BMI/age revealed an expected link with overweight and obesity status.

The Table 2 is the repartition within the cohort studied following anthropometric and cardiometablic data. Similarly, BMI and BMI/Age showed a significant association with IR. Regarding cardiometabolic aspects, all parameters showed a significant association with IR but Diastolic blood pressure.

The Table 3 is a relative prediction of developing IR, following risk factors. Obesity and a high Fat mass index are significantly associated with IR.

The Table 4 is the multivariate regression model of the identified risk factors in the Peruvian adolescents population. This table only represents the female subgroup, adding low physical activity to the previous associations.

The Discussion part summarizes the results obtained with emphasis on the associations of BMI and cardiometabolic parameters. The independence of physical inactivity and high fat mass index should be tested and reviewed.

Thank you for the observation.

The methodology section has been expanded in response to your comments.

**Figure 1 is a ROC curve to determine the optimal cut-off. This figure should be repositioned in the text as the starting element.**

Thank you for the observation.

We have positioned Figure 1 as the first result in the Results section.

**Results**

The HOMA-IR cutoff identified was 3.9, yielding a sensitivity of 72.4% and a specificity of 75.4%. The area under the ROC curve was 0.79 (95% CI: 0.69–0.88) (Figure 1).

See lines: 195-196

**Figure 2 is the mean prevalence of the risk factors mentioned earlier within IR/not IR individuals. This figure should be repositioned in the text with a few words of introduction and purpose.**

We have positioned Figure 2 as the third result in the Results section, following Figure 1 and Table 1.

**Some Typo :
Line 44 the end of the sentence is missing**

Thank you for the observation.

The missing information has been added.

excess weight at infancy and FHCD.

See lines: 44

**Line 57 mails -> males**

Thank you for the observation.

The missing information has been added.

% in males and

See lines: 57

**Line 75, 150 -> al. —> italic**

Thank you for the observation.

The missing information has been added.

ped by Matthews *et al.* and this

loped by Kowalski *et al*, 1997 and

See lines: 75; 148

**Table 2 Cardio-metabolic —> Cardiometabolic**

Thank you for the observation.

The indicated issue has been corrected.

**Line 288 y —> years old**
Thank you for the observation.

The missing information has been added.

adolescents (7–14 years old); in both sexes

See lines: 268

**And Sens :
Line 67 a reference is lacking linking hyperglycemia to hypertension at last**

The reference has been added.

Sowers, J. R., Standley, P. R., Ram, J. L., Jacober, S., Simpson, L., & Rose, K. (1993). Hyperinsulinemia, insulin resistance, and hyperglycemia: contributing factors in the pathogenesis of hypertension and atherosclerosis. *American journal of hypertension*, *6*(7 Pt 2), 260S–270S. <https://doi.org/10.1093/ajh/6.7.260s>

See lines: 68

**Line 76 « to the best of our knowledge » —> rephrase**

Thank you for the clarification. The wording of the sentence has been revised as follows:

Nonetheless, no studies in Peruvian adolescents have proposed specific cut-off points for HOMA-IR classification.

See lines: 79-80

**Table 1 n = 371?**
Yes, there was an error in the description of the total sample in the Methods section. The correct sample size is 371 adolescents. However, only the variables Fat Free Mass Index and Fat Mass Index in Tables 2 and 3 include data from 349 participants. We have clarified this in the *Study Design and Population* section to avoid confusion.

For fat-free mass (FFM) and fat-free mass index (FFMI), data were available for 349 participants; thus, related bivariate analyses used this subsample, while others used the full sample (n = 371).

See lines: 116-117

**As a summary, authors should clarify their study, first with a revision of the order of the figures/tables and their introduction/description in the manuscript. And then with a sequential discussion of theses results. Several aspects could be compared to recent results from the literature (Lee et al. 2023, Lozano et al. 2022, Rocca-Nacion et al. 2022, Zelada et al. 2016).**

Following your suggestion, we have carefully integrated the three recommended articles (Lee et al. 2023; Lozano et al., 2022; Rocca--Nación et al., 2022) into the manuscript. These references have been incorporated in a sequential and coherent manner within the Discussion, strengthening both the conceptual framework and the interpretation of our findings. As a result, the overall flow, depth, and clarity of the Discussion have been substantially improved.

See lines: 251-255; 257-261; 269-272

7. PLOS authors have the option to publish the peer review history of their article ([what does this mean?](https://track.editorialmanager.com/CL0/https:%2F%2Fjournals.plos.org%2Fplosone%2Fs%2Feditorial-and-peer-review-process%23loc-peer-review-history/1/010f019ce586750d-8cc3a999-90ed-4489-86cd-6a8d2b3e3230-000000/kAPbU0jFGnNnDlPzrwZjZKC_L_mdpoYE3f2tdVbL9a8=254)). If published, this will include your full peer review and any attached files.

If you choose “no”, your identity will remain anonymous but your review may still be made public.

**Do you want your identity to be public for this peer review?** For information about this choice, including consent withdrawal, please see our [Privacy Policy](https://track.editorialmanager.com/CL0/https:%2F%2Fwww.plos.org%2Fprivacy-policy/1/010f019ce586750d-8cc3a999-90ed-4489-86cd-6a8d2b3e3230-000000/GLWoinWnXUh5chulqVhPgVQ7UhcvHD9xc_kVm2om620=254).

Reviewer #1: No

Reviewer #2: No

Reviewer #5: No

[NOTE: If reviewer comments were submitted as an attachment file, they will be attached to this email and accessible via the submission site. Please log into your account, locate the manuscript record, and check for the action link "View Attachments". If this link does not appear, there are no attachment files.]

To ensure your figures meet our technical requirements, please review our figure guidelines: <https://journals.plos.org/plosone/s/figures>

You may also use PLOS’s free figure tool, NAAS, to help you prepare publication quality figures: <https://journals.plos.org/plosone/s/figures#loc-tools-for-figure-preparation>.

NAAS will assess whether your figures meet our technical requirements by comparing each figure against our figure specifications.
