# Supplementary material for: Identification of a HOMA-IR cut-off point for cardiometabolic risk and modifiable risk factors in peruvian adolescents
Source: PLoS One. 2026 Jul 10;21(7):e0351139. doi: 10.1371/journal.pone.0351139 (PMC13354073; doi:10.1371/journal.pone.0351139)
Supplement: Attachment — Submitted filename: Response Letter 11 05 26.docx [file pone.0351139.s007.docx]

PONE-D-25-02583R1

IDENTIFICATION OF A HOMA-IR CUT-OFF POINT FOR CARDIOMETABOLIC RISK AND MODIFIABLE RISK FACTORS IN PERUVIAN ADOLESCENTS

PLOS One


Reviewers' comments:

**1. Abstract Sample Size Inconsistency**: In response to Reviewer #2 regarding sample size confusion, the authors clarified in the main text that the total sample is 371, with a sub-sample of 349 used specifically for FFM/FFMI analyses. Unfortunately, the authors neglected to update the Abstract, which still states: "A sample of 349 adolescents...". The Abstract must be updated to reflect the full cohort of 371.

Thank you for the observation. We have corrected the error.

See lines: 33

**2. Abstract Diagnostic Overstatement:** The authors agreed to remove the subjective descriptor "high" when referring to sensitivity and specificity, modifying the Discussion accordingly. However, this overstatement was left intact in the Abstract, which still reads: "...with high sensitivity (72.4%) and specificity (75.4%).". This must be amended to match the neutral tone of the main text.

Thank you for the observation. The issue noted has been removed.

See lines: 40

**3. Scientific Typographical Error in Table 1:** While the authors successfully corrected the HOMA IR formula units in the Methods section (Lines 170-172) to µU/mL and mmol/L, the footnote for Table 1 contains a major scientific typo. It defines the calculation using fasting insulin in µU/L and fasting glucose in nmol/L. These units are incorrect for a divisor of 22.5 and must be corrected to match the main text.

Thank you for the observation. We have added the correct sample.

See table 1.
