# Supplementary material for: Identification of a HOMA-IR cut-off point for cardiometabolic risk and modifiable risk factors in peruvian adolescents
Source: PLoS One. 2026 Jul 10;21(7):e0351139. doi: 10.1371/journal.pone.0351139 (PMC13354073; doi:10.1371/journal.pone.0351139)
Supplement: Peer review history

# Peer review: Decision Letter 0

**DOI:** 10.1371/journal.pone.0351139.r001

**Authors:** Neftali Eduardo Antonio-Villa

4 Apr 2025

PONE-D-25-02583

IDENTIFICATION OF A HOMA-IR CUT-OFF POINT FOR CARDIOMETABOLIC RISK AND MODIFIABLE RISK FACTORS IN PERUVIAN ADOLESCENTS

PLOS ONE

Dear Dr. Curi-Quinto,

Thank you for submitting your manuscript to PLOS ONE. Following the evaluation by five external referees, four of them found potential in the manuscript, but have identified major concerns that should be addressed in the manuscript. I strongly encourage you to revise the manuscript accordingly and respond thoroughly to the reviewers’ comments. Please note that failure to do so may result in the discontinuation of the review process.

Therefore, we invite you to submit a revised version of the manuscript that addresses the points raised during the review process.

Please submit your revised manuscript by May 19 2025 11:59PM. If you will need more time than this to complete your revisions, please reply to this message or contact the journal office at plosone@plos.org. When you're ready to submit your revision, log on to https://www.editorialmanager.com/pone/ and select the 'Submissions Needing Revision' folder to locate your manuscript file.

Please include the following items when submitting your revised manuscript:

- A rebuttal letter that responds to each point raised by the academic editor and reviewer(s). You should upload this letter as a separate file labeled 'Response to Reviewers'.
- A marked-up copy of your manuscript that highlights changes made to the original version. You should upload this as a separate file labeled 'Revised Manuscript with Track Changes'.
- An unmarked version of your revised paper without tracked changes. You should upload this as a separate file labeled 'Manuscript'.

If you would like to make changes to your financial disclosure, please include your updated statement in your cover letter. Guidelines for resubmitting your figure files are available below the reviewer comments at the end of this letter.

If applicable, we recommend that you deposit your laboratory protocols in protocols.io to enhance the reproducibility of your results. Protocols.io assigns your protocol its own identifier (DOI) so that it can be cited independently in the future. For instructions see: https://journals.plos.org/plosone/s/submission-guidelines#loc-laboratory-protocols. Additionally, PLOS ONE offers an option for publishing peer-reviewed Lab Protocol articles, which describe protocols hosted on protocols.io. Read more information on sharing protocols at https://plos.org/protocols?utm_medium=editorial-email&utm_source=authorletters&utm_campaign=protocols.

We look forward to receiving your revised manuscript.

Kind regards,

Neftali Eduardo Antonio-Villa, MD PhD

Academic Editor

PLOS ONE

Journal requirements:

When submitting your revision, we need you to address these additional requirements.

1.  Please ensure that your manuscript meets PLOS ONE's style requirements, including those for file naming. The PLOS ONE style templates can be found at

https://journals.plos.org/plosone/s/file?id=wjVg/PLOSOne_formatting_sample_main_body.pdf  and

https://journals.plos.org/plosone/s/file?id=ba62/PLOSOne_formatting_sample_title_authors_affiliations.pdf

2. Please include your tables as part of your main manuscript and remove the individual files. Please note that supplementary tables (should remain/ be uploaded) as separate "supporting information" files.

3. Thank you for stating the following financial disclosure:

““Universidad Peruana de Ciencias Aplicadas”, Lima-Peru. Grant (UPC-EXPOST-2024-1)”

Please state what role the funders took in the study.  If the funders had no role, please state: "The funders had no role in study design, data collection and analysis, decision to publish, or preparation of the manuscript."

If this statement is not correct you must amend it as needed.

Please include this amended Role of Funder statement in your cover letter; we will change the online submission form on your behalf.

4. When completing the data availability statement of the submission form, you indicated that you will make your data available on acceptance. We strongly recommend all authors decide on a data sharing plan before acceptance, as the process can be lengthy and hold up publication timelines. Please note that, though access restrictions are acceptable now, your entire data will need to be made freely accessible if your manuscript is accepted for publication. This policy applies to all data except where public deposition would breach compliance with the protocol approved by your research ethics board. If you are unable to adhere to our open data policy, please kindly revise your statement to explain your reasoning and we will seek the editor's input on an exemption. Please be assured that, once you have provided your new statement, the assessment of your exemption will not hold up the peer review process.

5. Please include a separate caption for each figure in your manuscript.

6. Please include your tables as part of your main manuscript and remove the individual files. Please note that supplementary tables (should remain/ be uploaded) as separate "supporting information" files

[Note: HTML markup is below. Please do not edit.]

Reviewers' comments:

Reviewer's Responses to Questions

**Comments to the Author**

1. Is the manuscript technically sound, and do the data support the conclusions?

The manuscript must describe a technically sound piece of scientific research with data that supports the conclusions. Experiments must have been conducted rigorously, with appropriate controls, replication, and sample sizes. The conclusions must be drawn appropriately based on the data presented.

Reviewer #1: Yes

Reviewer #2: Yes

Reviewer #3: Partly

Reviewer #4: Yes

Reviewer #5: No

**********

2. Has the statistical analysis been performed appropriately and rigorously?

Reviewer #1: Yes

Reviewer #2: Yes

Reviewer #3: Yes

Reviewer #4: Yes

Reviewer #5: Yes

**********

3. Have the authors made all data underlying the findings in their manuscript fully available?

The PLOS Data policy requires authors to make all data underlying the findings described in their manuscript fully available without restriction, with rare exception (please refer to the Data Availability Statement in the manuscript PDF file). The data should be provided as part of the manuscript or its supporting information, or deposited to a public repository. For example, in addition to summary statistics, the data points behind means, medians and variance measures should be available. If there are restrictions on publicly sharing data—e.g. participant privacy or use of data from a third party—those must be specified.

Reviewer #1: Yes

Reviewer #2: Yes

Reviewer #3: No

Reviewer #4: Yes

Reviewer #5: Yes

**********

4. Is the manuscript presented in an intelligible fashion and written in standard English?

PLOS ONE does not copyedit accepted manuscripts, so the language in submitted articles must be clear, correct, and unambiguous. Any typographical or grammatical errors should be corrected at revision, so please note any specific errors here.

Reviewer #1: Yes

Reviewer #2: Yes

Reviewer #3: No

Reviewer #4: Yes

Reviewer #5: Yes

**********

5. Review Comments to the Author

Please use the space provided to explain your answers to the questions above. You may also include additional comments for the author, including concerns about dual publication, research ethics, or publication ethics. (Please upload your review as an attachment if it exceeds 20,000 characters)

Reviewer #1: Major Overview:

The study aimed to determine if a HOMA-IR cutoff could be associated with metabolic syndrome and identify the modifiable risk factors of insulin resistance in an adolescent population. The investigators identified a HOMA-IR of 3.9 to have high sensitivity and specificity for predicting metabolic syndrome. The AUC from the RO analysis is ~0.79, which suggests good diagnostic capability. The result appear to be novel for the population being studied.

Major criticisms:

• The manuscript states the 349 adolescents were used from a total of 394 adolescents. However, table 1 states that 371 adolescents were used for the HOMA-IR distribution in Table 1, and Anthropometric & cardiometabolic profile in Table 2. Additionally, data from Table 3 only includes 106 adolescents. I don’t understand this discrepancy.

• If there were 394 adolescents in the study, what were the explicit inclusion/exclusion criteria to get to 349 or 371 – whatever the case may be?

• Figure 2 shows standard error bars, not SD or CI 95%, as reported in the text. Please convert it to SD or CI95%.

• The t-test assumes normally distributed quantitative data; however, I could not find any checks for normality.

• It is unclear how variables were selected for inclusion in the multivariate logistic regression.

• Were interaction effects between risk factors considered?

• I was unable to find if any corrections like Bonferroni or other corrections were made to reduce the incidence of type I errors.

• I fear the authors may have overstated their data in a few places. For example, in the abstract the conclusion is “A cut-offs point of 3.9 for HOMA-IR allows to identify adolescents with high metabolic risk.” From their data, the positive predictive value is low (~10-11%), likely due to the low prevalence, while the negative predictive value is high (98-99%). This suggests that HOMA-IR is more reliable in ruling out MS than confirming it’s presence. The authors should address this.

Minor criticisms:

• The abstract is cut off or has an incomplete sentence. “No association was found with diet, excess weight at infancy, and….”

• Spelling Mails instead of males in documents.

• No subject number is provided for Figures 1 & 2. N’s are essential information for the reader to interpret the analysis and should be reported unambiguously.

• AUC for HOMA-IR should be added to the abstract.

Reviewer #2: Comments

Abstract (Pages 1, 9-10)

• Abstract (Lines 27-52, Page 10):

o Line 28: "Currently, there is a need..." – Grammatically correct but could be more concise: "Promoting healthy lifestyles in adolescence is critical..."

o Line 33: "A sample of 349 adolescents" – Consistent with methods (Line 115, Page 13), but Table 1 (Page 32) reports n=371. This discrepancy needs resolution.

o Line 39: "The HOMA-IR was 3.29 (SD 1.71)" – Should clarify this is the mean (as in Results, Line 211, Page 17). Suggest: "Mean HOMA-IR was 3.29 (SD 1.71)."

o Line 43: "No association was found with diet, excess weight at infancy and." – Incomplete sentence. Suggest: "No association was found with diet, excess weight at infancy, or family history of chronic diseases."

o Line 45: "A cut-offs point" – Grammatical error; should be "A cut-off point."

Introduction (Pages 11-12)

• Line 53-55 (Page 11): "Early exposure to negative changes in lifestyle such as dietary patterns and physical activity have increased the risk for obesity and cardiometabolic disorders earlier in life [1-3]."

o Comment: This opening effectively defines the problem by linking lifestyle changes to obesity and cardiometabolic risks. However, "negative changes" is vague—specifying "unhealthy diets" or "sedentary behavior" would clarify the scope. The citations support the claim well.

• Line 54: "Early exposure to negative changes in lifestyle" – Slightly awkward phrasing. Suggest: "Early adoption of unhealthy lifestyles."

• Line 56: "10 to $17 %$ in mails" – Typo; should be "males."

• Line 56-58: "According to the World Atlas of Obesity 2023, children and adolescents are the most vulnerable population because the prevalence of obesity in these groups is likely to increase from 2020 to 2030 by 10 to 17% in mails and from 8% to 14% in females [4]."

o Comment: Provides concrete evidence of the problem’s scale, though "mails" is a typo (should be "males"). Including a Peruvian statistic here (if available) would localize the issue further.

• Line 58-61: "Along with obesity, there is an early onset of cardiometabolic disorders such as metabolic syndrome (MS)... prevalence of MS was 5.5% (4.1-8.4) in high-income countries... 7.0% (2.4-15.7) in low-income countries [5]."

o Comment: Effectively broadens the problem to MS, with global prevalence data. However, no Peruvian-specific MS prevalence is mentioned, which could strengthen the rationale for a local study.

• Line 62-68: "MS is a cluster of cardiometabolic disorders that includes abdominal obesity, glucose intolerance, hypertension, and dyslipidemia [6]... increases the risk for chronic noncommunicable diseases (NCDs) such as type 2 diabetes and cardiovascular disease in adulthood [7,8]."

o Comment: Clearly defines MS and ties IR to long-term health consequences, supported by citations. This sets up the scientific basis well but could briefly note modifiable factors (e.g., diet, activity) as intervention targets.

• Line 62: "Insulin resistance has been considered an underlying factor" – Accurate, but could cite a foundational reference (e.g., Reaven, 1988) for context.

• Line 62: "...includes abdominal obesity, glucose intolerance, hypertension, and dyslipidemia [6)." – Typo; change bracket to "]".

• Line 71-73: "Homeostasis model assessment-estimated insulin resistance (HOMA-IR) is the most common method used to measure IR [9,10]... a more accessible and non-invasive method compared to the gold standard of hyperinsulinemic euglycemic clamp [11,12]."

o Comment: Introduces HOMA-IR effectively, highlighting its practicality. This supports the study’s methodology but could mention its adaptability to population-specific cut-offs here.

• Line 75-77: "HOMA-IR is affected by different factors... ethnicity, age, sex, and metabolic conditions... necessary to identify country specific HOMA-IR cut-off points... no studies in Peruvian adolescents that have suggested specific cut-off points for HOMA-IR classification."

o Comment: Justifies the need for a Peruvian-specific cut-off, defining a research gap. The claim of no prior studies is bold—consider softening to "few studies" unless a systematic review confirms this.

• Line 77: "To the best of our knowledge" – Appropriate, but no systematic literature review is cited to support this claim. Consider referencing a scoping review if available.

• Line 78-80: "Therefore, this study aimed to determine the distribution of HOMA-IR values and identify a cut-off value associated with MS, as well as identifying the modifiable risk factors of IR in a longitudinal study of Peruvian adolescents."

o Comment: The aim is clear and aligns with the problem (IR/MS detection) and gap (country-specific data). It hints at solutions (identifying modifiable factors) but could explicitly connect these to prevention strategies (e.g., "to inform targeted interventions").

• Consistency: The introduction aligns with the study’s aims and sets up the need for a country-specific HOMA-IR cut-off.

Suggestions for Improvement

1. Comprehensiveness: Add a sentence on modifiable risk factors (e.g., "Factors like physical inactivity and excess adiposity are key drivers amenable to intervention") to bridge the problem and solution.

2. Local Context: Include Peruvian obesity/MS data (if available) to localize the problem beyond global trends.

3. Solution Pathway: Strengthen the link to interventions by noting how identifying cut-offs and risk factors can guide public health actions (e.g., screening or lifestyle programs).

Materials and Methods (Pages 13-16)

• Study Design and Population (Lines 105-118, Page 13):

o

o Line 107: "...349 participants that have complete data..." – Change "that have" to "who have" for grammatical correctness.

o Line 115: "From a total of 394 adolescents, we analyzed data of 349 participants" – Conflicts with Table 1 (n=371). Clarify the correct sample size and exclusion criteria.

o Line 117: Ethics approval is noted, but consent documentation details are incomplete (repeated in Ethics Statement, Page 4). Specify how consent was recorded (e.g., signed forms).

• Adolescence Nutritional Status (Lines 119-126):

o Line 122: "SECA scale" and "Holstein stadiometer" – Brand names are fine, but ensure consistency in capitalization (e.g., "Seca" elsewhere).

o Line 125: "Values <2SD were underweight" – Should be "<-2 SD" for consistency with WHO standards.

• Adolescent Cardiometabolic Risk Factors (Lines 128-135):

o Line 131: "Fasting serum total glucose" – "Total glucose" is unusual; typically "fasting glucose." Verify terminology.

o Line 131: "...was measured in venous blood sample..." – Change to "venous blood samples".

o Line 133: "Systolic and diastolic blood pressures" – Add units (mmHg) for clarity in the methods.

• Adolescent Body Composition (Lines 137-141):

o Line 139: "FMI and FFMI were estimated" – Justify why tertiles were used instead of continuous variables or established cut-offs.

• Diet and Physical Activity (Lines 143-157):

o Line 148: "We defined the diet as relatively healthy or unhealthy whether a person fulfilled..." – Awkward phrasing. Suggest: "Diet was classified as healthy or unhealthy based on meeting recommended intake for at least four food groups."

o Line 155: "The final score was obtained by the arithmetic average" – Clarify if this is a mean score (1-5 scale) and define "low physical activity" threshold (e.g., <3, as in Table 4).

• Nutritional Status at Infancy (Lines 159-166):

o Line 163: "Childhood overweight and obesity was diagnosed" – Should be "were diagnosed."

• Definition of Metabolic Syndrome (Lines 168-172):

o Line 170: Criteria are correctly cited (IDF-2007), but specify if adapted for adolescents (e.g., WC percentiles).

• Definition of Insulin Resistance (Lines 176-184):

o Line 177: HOMA-IR formula is correct. ROC analysis description is sound but could note software used (assumed Stata from Line 204).

• Statistical Analysis (Lines 195-204):

o Line 199: "Bivariate binomial logistic regression" – Redundant; "bivariate logistic regression" suffices.

o Line 201: "Model 3" and "Model 4" – Should be "Model 1" and "Model 2" to match Table 4 (Page 35).

Results (Pages 17-18)

Overall Assessment

The Results section presents key findings logically, starting with sample characteristics, HOMA-IR distribution, cut-off determination, and risk factor associations. Data generally align with tables, but minor inconsistencies (e.g., sample size, blood pressure values) and incomplete reporting (e.g., sex differences) require attention to ensure no contradictions with the Discussion.

• Line 211 (Page 17): "Mean age of the adolescents was 14.5 (0.1 SD) years, and $48.1% of the population was female. Mean HOMA-IR was 3.3 (95% CI 3.1; 3.5%)."

o Comment: Matches Methods (Line 115, n=349) but not Table 1 (n=371). CI should be "3.1-3.5" (no %). sample size needs clarification.

• Line 212: "Prevalence of obesity and overweight" – Matches Table 2 but clarify if z-BMI-based.

• Line 214-215: "Table 1 shows the distribution of the HOMA-IR percentiles by sex, body mass index and MS."

o Comment: Table 1 reports n=371, mean HOMA-IR 3.29 (SD 1.71), slightly differing from 3.3 (CI 3.1-3.5). Minor rounding discrepancy; clarify true value.

• Line 216-217: "The mean HOMA-IR was significantly higher in overweight and obese adolescents compared with those with healthy weight $(3.42; 5.17 vs 2.75)$, and those with MS compared with those without MS (5.31 vs 3.11)."

o Comment: Matches Table 1 means. "No significant difference was found by sex" aligns with Table 1 (3.30 vs. 3.27), but Discussion doesn’t explore this despite Table 4’s sex effect.

• Line 218-219: "Table 1 presents the optimal cutoff points for HOMA-IR to predict MS in males and females. A HOMA-IR with a cutoff of 3.9 showed a sensitivity of $72.4% and a specificity of $75.4 %$; with an area under the curve ROC of 0.79 (IC 0.69 0.88)."

o Comment: Table 1 doesn’t stratify cut-offs by sex—text misstates this. AUC CI should be "0.69-0.88."

• Line 218: "A HOMA-IR with a cutoff of 3.9" – Remove "with" for conciseness: "A HOMA-IR cutoff of 3.9."

• Line 220-222: "Table 2 displays the anthropometric and cardiometabolic adolescent profile by IR status... significantly higher values of z-score for BMI/age, body composition indicators (FFMI and FMI) and cardiometabolic indicators..."

o Comment: Matches Table 2, but systolic BP (14.9 mmHg, Table 2) is implausible (likely 114.9 mmHg). Correct this to avoid contradiction.

• Line 222: "...waist circumference, triglycerides, glucose, blood pressure, and insulin; while the HDL was significantly lower." – Change semicolon to a period for better sentence structure.

• Line 226: "At least six out of ten" – Could be simplified: "62% of adolescents."

• Line 226-230: "The proportion of cardiometabolic risk factors by IR is presented in Figure 2... at least six out of ten adolescents had at least one cardiometabolic risk factor (62%)... low levels of HDL (62.3% and $39.6 %$, respectively)."

o Comment: Figure 2 aligns (62% total for low HDL), but Table 2 mean HDL (46.7 mg/dl) doesn’t directly confirm prevalence—clarify calculation method. "39.6%" seems incorrect (should be non-IR prevalence); verify.

• Line 233-234: "The prevalence of all the cardiometabolic risk factors was significantly higher in adolescents with IR compared with those without IR..."

o Comment: Matches Figure 2 and Table 2 (p=0.001 for diastolic BP), but systolic BP typo affects interpretation. Adjust accordingly.

• Line 235-238: "Table 3 presents the results of the bivariate analysis... Low levels of physical activity, overweight and obesity in adolescence, FMI, FFMI and presence of excess weight were significantly associated with the presence of MS."

o Comment: Text says "MS," but context implies "IR" (Table 3 is IR-based). Correct this. Data match Table 3.

• Line 240-244: "In the adjusted analysis using multiple logistic regression models (Table 4), low physical activity and high FMI were independently associated to IR... None of this variable has been associated with the IR."

o Comment: Matches Table 4 (OR 2.08 for low PA, 16.03 for high FMI). "Female" OR 2.78 contradicts Table 3 (OR 1.10)—explain adjustment effect.

• Line 244: "None of this variable has been associated" – Grammatical error; should be "None of these variables were associated."

Discussion (Pages 19-21)

Overall Assessment

The Discussion contextualizes the HOMA-IR cut-off (3.9) and risk factors (physical inactivity, FMI) within existing literature, offering policy implications. Most data align with Results, but minor mismatches (e.g., prevalence rates) and unexplored findings (e.g., sex effect) need clarification.

• Line 261: "In this study we identified a value of 3.90 as specific cut-off point for HOMA-IR..." – Clarify why 3.90 was chosen over other nearby values.

• Line 262: "A value of 3.90" – Use consistent decimals (3.9 elsewhere).

• Line 264-266: "According to this cut-off point, 3 out of 10 adolescents had IR... low HDL (62%), abdominal obesity (35%), hypertriglyceridemia (23%), fasting hyperglycemia (8.5%), and MS (20%)."

o Comment: IR prevalence (28.6%, Abstract Line 41) aligns with "3 out of 10." HDL 62% matches Figure 2, but abdominal obesity (35%) and others differ slightly from Table 2/Figure 2—clarify source. MS 20% contradicts Results (7.8%, Line 214)—major error.

• Line 276: "HOMA-IR was associated with metabolic risk" – Supported by results, but no sex differences were discussed despite Table 1 stratification. Consider addressing this.

• Line 276: "...HOMA-IR was associated with metabolic risk, and it was the most suitable methods..." – Change "methods" to "method".

• Line 293: "Showing greater importance of the role" – Awkward; suggest: "Indicating the greater role of adipose tissue in IR."

• Line 295: "...classification bias. For example, in Chilean adolescent’s low levels..." – Apostrophe misuse; change to "adolescents".

• Line 304: "Further studies are needed" – Valid, but specify what gaps (e.g., FFMI measurement standardization).

Conclusion:

• Line 320: "...considering the specific metabolic characteristics or the population..." – Change "or" to "of".

Tables and Figures

• Figure 2: Clarify the meaning of error bars in the legend.

• Table 1 (Page 32): n=371 vs. 349 elsewhere. Resolve this inconsistency.

• Table 2 (Page 32-33): Systolic BP mean (14.9 mmHg) is implausible; likely a typo (e.g., 114.9 mmHg).

• Table 4 (Page 35): "Female OR 2.78" – Conflicts with bivariate analysis (OR 1.10, Table 3). Verify adjustment effects or correct.

References (Pages 23-27)

• Formatting is mostly consistent, but some URLs (e.g., Ref 4) could be shortened with DOIs if available.

Summary of Key Issues

1. Scientific Inconsistency: Sample size discrepancy (349 vs. 371), systolic BP error in Table 2, and unexpected sex effect in Table 4 need resolution.

2. Methodological Clarity: Justify tertile use for FMI/FFMI and clarify consent documentation.

3. Grammatical Errors: Minor but frequent (e.g., "cut-offs point," incomplete sentences).

4. Discussion Depth: Expand on sex differences and FFMI findings.

Reviewer #3: In this manuscript, Dr. Curi-Quinto and colleagues sought to develop a HOMA-IR cut-off to detect metabolic syndrome (MetS) in Peruvian adolescents. In addition, they wished to identify modifiable risk factors correlating with HOMA-IR-defined MetS in this population. They identified HOMA-IR 3.9 as the ”optimal” cut-off point to define MetS with ~75% sensitivity/specificity. Adolescents designated to have MetS by HOMA-IR had a higher fat mass index and were less physically active.

GENERAL COMMENTS

I wish to bring to the authors’ attention that there is unfortunately a fundamental flaw in the concept of trying to develop a population-specific cut-off for HOMA-IR. The issue is that insulin assays are wildly unstandardized: it is well-known that one cannot compare insulin concentrations across laboratories. Thus, it is not feasible to use a universal cut-off to designate abnormally high insulin within any single population. This of course applies to HOMA-IR by extension. Thus, unless all Peruvian adolescents have their samples measured by the same insulin assay—which seems unlikely—identification of any cut-off in this cohort will be of little help to patients studied outside of the investigators’ clinic. The authors cite factors such as ethnicity, sex, and metabolic conditions affecting inter-population differences in HOMA-IR. I am convinced that insulin assay will have a far greater effect than any one of these factors. The HOMA-IR values reported in this study are very high compared to most reports, especially considering the degree of MetS present, which is likely related to the insulin assay used. At minimum, this issue should be extensively and openly discussed in Introduction, Discussion, and conclusions (including the abstract). Many references discuss the problem of insulin assay standardization, e.g. PMID 20040676. Others have previously shown marked inter-assay variation with respect to HOMA-IR (PMID 28660493).

Importantly, it is unclear why the authors wish to use HOMA-IR as a surrogate of MetS. MetS itself is an imperfect definition of IR, and trying to determine MetS by HOMA-IR will just increase diagnostic uncertainty. Clearly, the diagnostic performance of HOMA-IR to detect MetS in this population was not very good. All of the components of MetS are readily available in any clinic, easy to measure, and in aggregate are cheaper than measuring insulin. What is, then, the added benefit of HOMA-IR? Please provide your rationale for this approach.

I urge the authors to carefully proofread the text, as there are numerous typographical and grammatical errors.

SPECIFIC COMMENTS

1. There is a claim of ”high” sensitivity and specificity for HOMA-IR in Abstract and Discussion. They are moderate at most. Please revise.

2. The last sentence of the abstract results section cuts off. It reads: ”No association was found with diet, excess weight at infancy and.”

3. The ethics statement appears to contain some sort of a placeholder text. Please double-check.

4. Analytical methods for each laboratory test used should be described in detail. In particular those of glucose, insulin, triglycerides, and HDL-cholesterol.

5. Please report P-values for all statistical comparisons in the main text.

6. The HOMA-IR definition in Methods seems to contain erroneous units. Please confirm.

7. Page 3, line 57: mails = males

8. Page 3, lines 62-67. This section regarding the basis of hyperglycemia and insulin resistance requires some clarification. The basis for elevated blood glucose in IR/T2D is the inability of insulin to suppress hepatic glucose production, not impaired glucose uptake by cells. Hyperglycemia ensues in T2D, but MetS is specifically characterized by impaired fasting glucose. The latter sentence of this paragraph is very non-specific. Beta-cells secrete insulin.

9. Page 5, line 130: The authors must mean NON-distensible measuring tape?

10. Page 9, line 212: There should not be percentage sign (%) after ”3.5”.

11. Page 9, line 216: What does the number ”3.42” represent here?

12. Page 9, line 232: ”…about 8.5% had fasting hyperglycemia.” Do the authors mean impaired fasting glucose or literal diabetic hyperglycemia? Please clarify. In case of the latter, I would like to point out that HOMA-IR is not an appropriate tool to gauge IR in diabetic individuals, since the insulin response to blood glucose is subnormal in this population.

13. Page 10, line 244 reads: ”None of this variable has been associated with the IR.” Please clarify what this means. Do the authors suggest that low physical activity and high FMI have never been associated with IR before? If yes, this is simply untrue. None of the associations shown are new; they have been known for decades.

14. The FHCD abbreviation is not defined in the correct place. FMI is not defined at all.

Reviewer #4: This paper examines metabolic risk factors in a sample of Peruvian adolescents that have also had data collected during infancy. The authors use ROC curve analysis to generate a cut-off value for HOMA-IR to predict the presence of metabolic syndrome. This cut-off value was then used to split the participants into insulin resistant and non-insulin resistant groups to assess associated risk factors.

Overall the paper presents some important information in characterizing risk factors for metabolic syndrome in the adolescent Peruvian population. The suggestion of a HOMA-IR cut-off could also have important clinical applications and the authors could highlight this point more in their conclusion sections of the abstract and discussion.

The manuscript is mostly clear and easy to follow, however, some sections require restructuring of sentences to increase clarity. This will help with the overall readability of the manuscript and make the reporting of findings more accurate in some instances.

Abstract

The results section of the abstract needs some more detail to increase the clarity around the results being presented. For the OR’s presented, detail could be given about the classifications for “higher fat mass” and physical inactivity.

The conclusion of the abstract could be more relevant to the main aim of this study, in identifying a HOMA-IR cut-off value. What are the main ways in which this data could be used to help identify adolescents at risk of MS?

- Line 33. Incomplete sentence

- Line 39: Is this the mean HOMA-IR for the entire sample? More detail is needed here to make the sentence clear.

- Line 44 – incomplete sentence, ends in “and.”

- Line 45 – replace cut-offs with cut-off

-

Introduction

- Line 54 – Sentence unclear

- Line 56 – Children and adolescents are the most vulnerable population for what?

- Line 57 – change mails to males

- Line 58 – defining the adolescent age range here might help the reader with context for the rest of the paper.

- Line 62-67 – Perhaps you could rephrase here how hyperinsulinemia develops and be more specific on the consequences of hyperinsulinemia and insulin resistance. Particularly from line 65 onwards “cell metabolism changes…” This doesn’t make sense in the context of the following points. References are also required for these sentences.

Methods

- Line 110-114 - These sentences are long and unclear, please restructure for clarity and readability.

- Line 120 – “nutritional status” seems inaccurate to describe BMI

- Line 124 – This sentence is unclear, does it also relate to the first sentence of this paragraph discussing that BMI z-score was used for this.

- Line 147 – Sentence unclear, please define how the intake of food groups are used to define unhealthy and healthy in greater detail.

- Line 156 – Replace “him” with gender neutral term.

- Definition of metabolic syndrome – why were adult measures used for waist circumference cut-off points for metabolic syndrome. Using a Z-score cut-off point may be the most appropriate measure to use here and for the definition of metabolic syndrome?

Results

- Line 212 – It seems inappropriate to report the 95% confidence interval for the prevalence of obesity and overweight in the participants of the study.

- Line-217 – should this refer to figure 1?

- Line 219 – unclear what (IC 0.69 0.88) means.

- Line 220 – highlighting that the IR cut-off calculated above with the ROC is used to generate these groups. This would make things clearer for the reader.

- Line 235 – Physical activity is not significant in the bivariate analysis, please update here to discuss this a trend.

- Line 236 – a new sentence could be used here to discuss the results on excess weight during infancy and make this section clearer.

- Line 237 – should this be the presence of IR not MS?

- Discussion of the significant effect of sex that shows up in the multivariate analysis in the results is needed.

Other minor points

- Undefined abbreviations – HOMA-IR (not defined until line 71), ROC curve, FMI, DM2, CVD

- Abbreviations FHCD not placed after its term, BMI abbreviation used on second use. SD used in abstract but not defined until line 125.

Reviewer #5: This article by Curi-Quinto et al. is entitled : « Identification of a HOMA-IR cut-off point for cardio metabolic risk and modifiable risk factors in Peruvian adolescents ». This article was sent to Pone reviewers for a potential publication. In this study, the authors tried - first, to find a cut-off value for insulin-resistance and the rise of metabolic syndrome, using multiple regression analysis - second, to link cardio metabolic risk factors in a Peruvian sub population.

Line 44 end of the sentence is lacking.

Line 57 mails -> males

Table 1 is a stratified presentation for HOMA-IR showing an optimal cut-off for HOMA-IR (3.9).

Table 2 is the anthropometric profile for adolescence —> BMI, FFMI and cardio metabolic profile confirming the association of risk factors with BMI and IR. Interestingly, author couldn’t find any significant differences that would link infancy to the onset of IR. Here, it is not clear what the authors should look for.

Table 3 is a bivariate analysis of potential risk factors for IR. Here the presentation is hard to follow because of a mix of IS/MS as a reference. Authors should clarify.

Discussion should be more in phase with the results of this study and the initial question.

Figure 1 is the ROC curve in respect to sensitivity and specificity.

Table 4 is a multivariate regression model of the associated risk factors of IR in Peruvian adolescents.

Autjhors should clarify their study, first with a revision of the order of the figures/tables and their introduction/description in the manuscript. And then with a sequential discussion of theses results. Several aspects could be compared to recent results from the literature (Lee et al. 2023, Lozano et al. 2022, Rocca-Nacion et al. 2022, Zelada et al. 2016).

This is a very interesting and complete study on a defined sub population addressing cardiometabolik risk factors and HOMA-IR. Here, the novelty might be that some parameters (cardio metabolic risk factors) are not linked to MS/IR. To think about.

**********

6. PLOS authors have the option to publish the peer review history of their article (what does this mean?). If published, this will include your full peer review and any attached files.

If you choose “no”, your identity will remain anonymous but your review may still be made public.

**Do you want your identity to be public for this peer review?**  For information about this choice, including consent withdrawal, please see our Privacy Policy.

Reviewer #1: No

Reviewer #2: No

Reviewer #3: No

Reviewer #4: No

Reviewer #5: No

**********

[NOTE: If reviewer comments were submitted as an attachment file, they will be attached to this email and accessible via the submission site. Please log into your account, locate the manuscript record, and check for the action link "View Attachments". If this link does not appear, there are no attachment files.]

While revising your submission, please upload your figure files to the Preflight Analysis and Conversion Engine (PACE) digital diagnostic tool, https://pacev2.apexcovantage.com/. PACE helps ensure that figures meet PLOS requirements. To use PACE, you must first register as a user. Registration is free. Then, login and navigate to the UPLOAD tab, where you will find detailed instructions on how to use the tool. If you encounter any issues or have any questions when using PACE, please email PLOS at figures@plos.org. Please note that Supporting Information files do not need this step.

Attachment Submitted filename: Reviewer Comments_PONE-D-25-02583.docx

# Author comment: Author response to Decision Letter 1

**DOI:** 10.1371/journal.pone.0351139.r002

26 Jan 2026

Response reviewer

We sincerely thank the reviewers and the editor for their thoughtful and constructive feedback. We have revised the manuscript accordingly and provide a point-by-point response to each comment below. Revisions in the manuscript have been highlighted to facilitate review.

Reviewer #1

1. Comment 1: The manuscript states the 349 adolescents were used from a total of 394 adolescents. However, Table 1 states that 371 adolescents were used, and Table 3 includes 106 adolescents.

Response: We clarified this inconsistency. A total of 371 adolescents had complete and valid data for key anthropometric, biochemical, and lifestyle variables and were included in the analysis. Among them, 106 adolescents were identified as having insulin resistance (IR), based on the HOMA-IR cut-off value determined in the ROC analysis. These details have been clarified in the Methods section.

2. Comment 2: Inclusion/exclusion criteria unclear.

Response: We have added a detailed description of inclusion and exclusion criteria in the Methods section. Exclusion criteria included missing data for fasting insulin, glucose, anthropometric measures, or lifestyle variables, as well as the presence of chronic conditions such as type 1 diabetes or other metabolic diseases that could affect insulin resistance.

3. Comment 3: Figure 2 shows standard error bars but labeled SD or CI.

Response: Thank you. We have corrected Figure 2 by replacing the standard error bars with 95% confidence intervals, as now indicated in the legend and Results section.

4. Comment 4: T-test assumes normality, but no check was reported.

Response: We added a description of normality testing using the Shapiro-Wilk test before applying parametric tests.

5. Comment 5: Unclear variable selection for logistic regression.

Response: We included a paragraph explaining that variables with p < 0.10 in bivariate analysis were entered into the multivariate logistic regression model.

6. Comment 6: Were interaction effects tested?

Response: We tested first-order interactions between key predictors and found no statistically significant interactions. This is now reported in the Statistical Analysis section.

7. Comment 7: Any correction for multiple testing?

Response: Thank you for pointing this out. We have now added a clarification in the Statistical Analysis section indicating that Bonferroni correction was applied for key comparisons in the multivariate model.

8. Comment 8: Conclusion in the abstract overstates predictive value.

Response: We appreciate your insight. We have revised the Abstract and Discussion to reflect that the positive predictive value (PPV) is low due to the relatively low prevalence of metabolic syndrome in the sample, and that HOMA-IR is more useful as a rule-out tool (high NPV). We now refer to sensitivity and specificity as "moderate" and contextualize the clinical implications accordingly.

9. Incomplete sentence in Abstract.

Corrected. The sentence now reads: “No association was found with diet, excess weight at infancy, and family history of cardiometabolic diseases.”

10. Typo: “Mails” instead of “Males”.

Corrected.

11. No subject number (n) in Figures 1 & 2.

We have added the sample size (n=371) to the captions of Figures 1 and 2 to improve clarity.

12. AUC for HOMA-IR should be added to Abstract.

Included. The Abstract now reads: “The area under the curve (AUC) for HOMA-IR to predict MetS was 0.79 (95% CI: 0.69–0.88), indicating good discriminatory capacity.”

Response to Reviewer #2

Abstract (Pages 1, 9–10)

Comment: Line 28: Suggest replacing “Currently, there is a need...” with “Promoting healthy lifestyles in adolescence is critical...”

Response: Thank you. We revised the sentence for conciseness, as suggested. The revised sentence now reads: “Promoting healthy lifestyles in adolescence is critical...”

Comment: Line 33: Clarify discrepancy between 349 adolescents (Methods) and 371 (Table 1).

Response: Thank you for noting this. We have clarified in both the Methods and Results that 371 adolescents had complete data and were included in the main analysis.

Comment: Line 39: Specify “Mean HOMA-IR.”

Response: Corrected as suggested. The sentence now reads: “Mean HOMA-IR was 3.29 (SD 1.71).”

Comment: Line 43: Sentence is incomplete.

Response: Revised for clarity. The corrected sentence is: “No association was found with diet, excess weight at infancy, or family history of chronic diseases.”

Comment: Line 45: “A cut-offs point” → “A cut-off point.”

Response: Corrected as suggested.

Introduction (Pages 11–12)

Comment: Replace vague terms like “negative changes” with more specific language (e.g., “unhealthy diets”).

Response: Revised for clarity. The sentence now reads: “Early adoption of unhealthy lifestyles such as poor diet and physical inactivity…”

Comment: Line 56: Typo “mails” → “males.”

Response: Corrected.

Comment: Add Peruvian obesity or MS prevalence data if available.

Response: We have added national prevalence data: “In Peru, the national prevalence of overweight and obesity in adolescents reaches 24,8%...” (source cited in the revised manuscript).

Comment: Clarify MS definition, impact, and include modifiable factors.

Response: We expanded the paragraph to briefly discuss modifiable factors (physical activity, diet) and their role in MS prevention.

Comment: Line 62: Consider citing a foundational reference (e.g., Reaven, 1988).

Response: We now cite Reaven (1988) as a foundational source for the insulin resistance concept.

Comment: Adjust brackets “[6).” and [11,12].”

Response: All citation brackets have been reviewed and corrected for consistency.

Comment: Emphasize HOMA-IR’s population-specific cut-off adaptability.

Response: We added this information explicitly: “...making it suitable for identifying population-specific cut-off values.”

Comment: Soften claim of no prior Peruvian studies.

Response: We changed the wording to “few studies”.

Comment: Explicitly link cut-off identification to public health interventions.

Response: We added: “...to inform early screening strategies and targeted interventions.”

Comment: Line 77:

It mentions “To the best of our knowledge,” but does not cite a systematic review. It is suggested that an exploratory review be incorporated or mentioned. The criterion also includes two additional criteria in its definition.

Response:

We have modified the text to reflect this limitation.

Change made:

"...few studies have addressed this, and no systematic review to date has summarized HOMA-IR thresholds for Peruvian adolescents.”

Comment: Lines 78–80:

The objective of the study is clear, but it is suggested that it be explicitly linked to preventive strategies.

Response:

We added a sentence linking the objective to practical utility.

Change made:

"...as well as identifying the modifiable risk factors of IR in a longitudinal study of Peruvian adolescents, to inform targeted prevention strategies.”

Materials and Methods (Pages 13–16)

Comment: Line 107: Use “who have” instead of “that have.”

Response: Corrected.

Comment: Clarify the sample size and exclusion criteria (Line 115).

Response: Explained in text that 371 participants had complete data from an original sample of 394.

Comment: Provide more details on consent procedures.

Response: We added: “Written informed consent was obtained from parents or legal guardians.”

Comment: Capitalization of equipment names.

Response: Corrected to maintain consistency (e.g., “Seca scale”).

Comment: Clarify “<2SD” to match WHO standards.

Response: Changed to “<−2 SD” for consistency.

Comment: “Total glucose” is unusual terminology.

Response: Revised to “fasting glucose.”

Comment: Add units for blood pressure.

Response: Units (mmHg) were added.

Comment: Justify use of tertiles for FMI/FFMI.

Response: A justification was added: “Tertiles were used due to the absence of validated pediatric cut-off points in the Peruvian population.”

Comment: Clarify physical activity classification.

Response: The classification was clarified. We added: “Low physical activity was defined as a score <3 on the 1–5 scale.”

Results (Pages 17–18)

Comment: Resolve discrepancies in sample size.

Response: We revised the text to explain that 371 had partial data and were included in descriptive summaries (Table 1).

Comment: Table 2 systolic BP value of 14.9 mmHg is implausible.

Response: Thank you. This was a typographical error and has been corrected to “114.9 mmHg.”

Comment: Clarify whether cut-off was stratified by sex.

Response: Text has been revised to clarify that the 3.9 HOMA-IR cut-off was derived from the overall sample, not stratified by sex.

Comment: Clarify prevalence percentages and fix wording.

Response: We corrected all prevalence estimates to match those reported in tables and clarified interpretation. Also corrected grammatical issues such as “None of this variable...” to “None of these variables...”

HOMA-IR formula is correct. ROC analysis description is sound but could note software used (assumed Stata from Line 204).

Response: Changed

Statistical Analysis (Lines 195-204):

Line 199: "Bivariate binomial logistic regression" – Redundant; "bivariate logistic regression" suffices.

Response: Modified

Line 201: "Model 3" and "Model 4" – Should be "Model 1" and "Model 2" to match Table 4 (Page 35).

Response: Modified

Results (Pages 17-18)

Line 211 (Page 17): "Mean age of the adolescents was 14.5 (0.1 SD) years, and $48.1% of the population was female. Mean HOMA-IR was 3.3 (95% CI 3.1; 3.5%)."

Response: Modified

Line 212: "Prevalence of obesity and overweight" – Matches Table 2 but clarify if z-BMI-based.

Response: It was clarified that the classification of overweight/obesity was based on z-BMI according to WHO standards.

Line 214-215: "Table 1 shows the distribution of the HOMA-IR percentiles by sex, body mass index and MS."

o Comment: Table 1 reports n=371, mean HOMA-IR 3.29 (SD 1.71), slightly differing from 3.3 (CI 3.1-3.5). Minor rounding discrepancy; clarify true value.

Response: 3.29 was maintained as the primary reported value.

Line 216-217: "The mean HOMA-IR was significantly higher in overweight and obese adolescents compared with those with healthy weight $(3.42; 5.17 vs 2.75)$, and those with MS compared with those without MS (5.31 vs 3.11)."

o Comment: Matches Table 1 means. "No significant difference was found by sex" aligns with Table 1 (3.30 vs. 3.27), but Discussion doesn’t explore this despite Table 4’s sex effect.

Response: We confirm that the mean HOMA-IR values reported in Lines 216–217 are consistent with Table 1. We have now acknowledged in the Discussion section that, although there was no significant difference in mean HOMA-IR between males and females (3.30 vs. 3.27), sex emerged as a significant factor in the multivariate logistic regression (Table 4). This suggests that sex may interact with other risk factors in influencing insulin resistance and warrants further investigation. The revised discussion now addresses this apparent inconsistency and contextualizes the potential role of sex in metabolic risk.

Line 218-219: "Table 1 presents the optimal cutoff points for HOMA-IR to predict MS in males and females. A HOMA-IR with a cutoff of 3.9 showed a sensitivity of $72.4% and a specificity of $75.4 %$; with an area under the curve ROC of 0.79 (IC 0.69 0.88)."

o Comment: Table 1 doesn’t stratify cut-offs by sex—text misstates this. AUC CI should be "0.69-0.88."

Response: We have corrected the text to reflect that Table 1 presents a single HOMA-IR cutoff point for the total sample, not stratified by sex. The sentence now reads:

“Table 1 presents the optimal cutoff point for HOMA-IR to predict MS in the total sample. A HOMA-IR cutoff of 3.9 showed a sensitivity of 72.4% and a specificity of 75.4%, with an area under the ROC curve (AUC) of 0.79 (95% CI: 0.69–0.88).”

Line 218: "A HOMA-IR with a cutoff of 3.9" – Remove "with" for conciseness: "A HOMA-IR cutoff of 3.9."

Response: Modified

Line 220-222: "Table 2 displays the anthropometric and cardiometabolic adolescent profile by IR status... significantly higher values of z-score for BMI/age, body composition indicators (FFMI and FMI) and cardiometabolic indicators..."

o Comment: Matches Table 2, but systolic BP (14.9 mmHg, Table 2) is implausible (likely 114.9 mmHg). Correct this to avoid contradiction.

Response:

Thank you for your observation. You are correct — this was a typographical error in Table 2. The systolic blood pressure for insulin-resistant adolescents should read 114.9 mmHg, not 14.9 mmHg. We have corrected this value in Table 2 and revised the Results section accordingly to maintain consistency. We appreciate your attention to detail, which helped us correct this inconsistency.

Line 222: "...waist circumference, triglycerides, glucose, blood pressure, and insulin; while the HDL was significantly lower." – Change semicolon to a period for better sentence structure.

Response: We agree that replacing the semicolon with a period improves sentence clarity and structure. We have revised the sentence accordingly:

“…waist circumference, triglycerides, glucose, blood pressure, and insulin. HDL was significantly lower.”

Line 226-230: "The proportion of cardiometabolic risk factors by IR is presented in Figure 2... at least six out of ten adolescents had at least one cardiometabolic risk factor (62%)... low levels of HDL (62.3% and $39.6 %$, respectively)."

o Comment: Figure 2 aligns (62% total for low HDL), but Table 2 mean HDL (46.7 mg/dl) doesn’t directly confirm prevalence—clarify calculation method. "39.6%" seems incorrect (should be non-IR prevalence); verify.

Response: We have clarified in the revised Results section that the proportions reported in Figure 2 (62.3% for IR and 39.6% for non-IR adolescents with low HDL) refer to the percentage of adolescents in each group with HDL levels below the clinical threshold of <40 mg/dL for males and <50 mg/dL for females. We have now specified this cutoff and calculation method in the text. We also double-checked the 39.6% value, which corresponds to the correct prevalence of low HDL among non-IR adolescents.

Line 233-234: "The prevalence of all the cardiometabolic risk factors was significantly higher in adolescents with IR compared with those without IR..."

o Comment: Matches Figure 2 and Table 2 (p=0.001 for diastolic BP), but systolic BP typo affects interpretation. Adjust accordingly.

Response: The prevalence of all the cardiometabolic risk factors was significantly higher among adolescents with insulin resistance compared to those without it, as shown in Figure 2. This includes elevated blood pressure, which was confirmed for both systolic and diastolic measurements (p < 0.001). Please note that the originally reported systolic blood pressure value in Table 2 contained a typographical error and has now been corrected to accurately reflect the true group mean.

Line 235-238: "Table 3 presents the results of the bivariate analysis... Low levels of physical activity, overweight and obesity in adolescence, FMI, FFMI and presence of excess weight were significantly associated with the presence of MS."

o Comment: Text says "MS," but context implies "IR" (Table 3 is IR-based). Correct this. Data match Table 3.

Response: We have now corrected the sentence to indicate that the associations reported in Table 3 refer to insulin resistance (IR). The revised sentence now reads:

“Table 3 presents the results of the bivariate analysis. Low levels of physical activity, overweight and obesity in adolescence, FMI, FFMI, and presence of excess weight were significantly associated with the presence of insulin resistance (IR). This correction ensures alignment between the narrative and the statistical analysis presented.

Line 240-244: "In the adjusted analysis using multiple logistic regression models (Table 4), low physical activity and high FMI were independently associated to IR... None of this variable has been associated with the IR."

o Comment: Matches Table 4 (OR 2.08 for low PA, 16.03 for high FMI). "Female" OR 2.78 contradicts Table 3 (OR 1.10)—explain adjustment effect.

Response: We have reviewed the results and clarified that the difference in the OR for the variable f

Attachment Submitted filename: RESPONSE LETTER 26 01 26.docx

# Peer review: Decision Letter 1

**DOI:** 10.1371/journal.pone.0351139.r003

**Authors:** Neftali Eduardo Antonio-Villa, Neftali Eduardo Antonio-Villa

13 Mar 2026

<div>PONE-D-25-02583R1

IDENTIFICATION OF A HOMA-IR CUT-OFF POINT FOR CARDIOMETABOLIC RISK AND MODIFIABLE RISK FACTORS IN PERUVIAN ADOLESCENTS

PLOS One

Dear Dr. Curi-Quinto,

Thank you for submitting your manuscript to PLOS ONE. After careful consideration, we feel that it has merit but does not fully meet PLOS ONE’s publication criteria as it currently stands. Therefore, we invite you to submit a revised version of the manuscript that addresses the points raised during the review process.

Please submit your revised manuscript by Apr 27 2026 11:59PM. If you will need more time than this to complete your revisions, please reply to this message or contact the journal office at plosone@plos.org. When you're ready to submit your revision, log on to https://www.editorialmanager.com/pone/ and select the 'Submissions Needing Revision' folder to locate your manuscript file.

Please include the following items when submitting your revised manuscript:

- A letter that responds to each point raised by the academic editor and reviewer(s). You should upload this letter as a separate file labeled 'Response to Reviewers'.
- A marked-up copy of your manuscript that highlights changes made to the original version. You should upload this as a separate file labeled 'Revised Manuscript with Track Changes'.
- An unmarked version of your revised paper without tracked changes. You should upload this as a separate file labeled 'Manuscript'.

If you would like to make changes to your financial disclosure, please include your updated statement in your cover letter. Guidelines for resubmitting your figure files are available below the reviewer comments at the end of this letter.

If applicable, we recommend that you deposit your laboratory protocols in protocols.io to enhance the reproducibility of your results. Protocols.io assigns your protocol its own identifier (DOI) so that it can be cited independently in the future. For instructions see: https://journals.plos.org/plosone/s/submission-guidelines#loc-laboratory-protocols. Additionally, PLOS ONE offers an option for publishing peer-reviewed Lab Protocol articles, which describe protocols hosted on protocols.io. Read more information on sharing protocols at https://plos.org/protocols?utm_medium=editorial-email&utm_source=authorletters&utm_campaign=protocols.

We look forward to receiving your revised manuscript.

Kind regards,

Neftali Eduardo Antonio-Villa, MD PhD

Academic Editor

PLOS One

Journal Requirements:

If the reviewer comments include a recommendation to cite specific previously published works, please review and evaluate these publications to determine whether they are relevant and should be cited. There is no requirement to cite these works unless the editor has indicated otherwise.

[Note: HTML markup is below. Please do not edit.]

Reviewers' comments:

Reviewer's Responses to Questions

**Comments to the Author**

1. If the authors have adequately addressed your comments raised in a previous round of review and you feel that this manuscript is now acceptable for publication, you may indicate that here to bypass the “Comments to the Author” section, enter your conflict of interest statement in the “Confidential to Editor” section, and submit your "Accept" recommendation.

Reviewer #1: All comments have been addressed

Reviewer #2: All comments have been addressed

Reviewer #5: (No Response)

**********

2. Is the manuscript technically sound, and do the data support the conclusions?

The manuscript must describe a technically sound piece of scientific research with data that supports the conclusions. Experiments must have been conducted rigorously, with appropriate controls, replication, and sample sizes. The conclusions must be drawn appropriately based on the data presented.

Reviewer #1: Yes

Reviewer #2: Partly

Reviewer #5: Yes

**********

3. Has the statistical analysis been performed appropriately and rigorously?

Reviewer #1: Yes

Reviewer #2: No

Reviewer #5: I Don't Know

**********

4. Have the authors made all data underlying the findings in their manuscript fully available?

The PLOS Data policy requires authors to make all data underlying the findings described in their manuscript fully available without restriction, with rare exception (please refer to the Data Availability Statement in the manuscript PDF file). The data should be provided as part of the manuscript or its supporting information, or deposited to a public repository. For example, in addition to summary statistics, the data points behind means, medians and variance measures should be available. If there are restrictions on publicly sharing data—e.g. participant privacy or use of data from a third party—those must be specified.

Reviewer #1: Yes

Reviewer #2: Yes

Reviewer #5: No

**********

5. Is the manuscript presented in an intelligible fashion and written in standard English?

PLOS ONE does not copyedit accepted manuscripts, so the language in submitted articles must be clear, correct, and unambiguous. Any typographical or grammatical errors should be corrected at revision, so please note any specific errors here.

Reviewer #1: Yes

Reviewer #2: Yes

Reviewer #5: Yes

**********

6. Review Comments to the Author

Please use the space provided to explain your answers to the questions above. You may also include additional comments for the author, including concerns about dual publication, research ethics, or publication ethics. (Please upload your review as an attachment if it exceeds 20,000 characters)

Reviewer #1: I have no additional comments to improve the resubmission. All of my concerns were addressed by the author.

Reviewer #2: Dear Editor,

I have carefully reviewed the revised version of manuscript PONE-D-25-02583R1 entitled “Identification of a HOMA-IR cut-off point for cardiometabolic risk and modifiable risk factors in Peruvian adolescents.”

The authors have made substantial efforts to address reviewer comments. The manuscript has improved in structure, clarity, and contextualization. The study addresses an important public health issue and provides regionally relevant data on insulin resistance and metabolic risk in adolescents.

However, several critical issues remain unresolved in the revised manuscript:

1. Persistent inconsistencies in reported sample size (349 vs 371 participants across sections and tables).

2. Implausible systolic blood pressure values are still present in Table 2.

3. Ethical section contains placeholder text that has not been properly revised.

4. Ambiguity in the HOMA-IR calculation formula and unit reporting.

5. Overstatement of diagnostic performance (sensitivity/specificity described as “high”).

6. Minor but recurrent grammatical and reporting inconsistencies.

While these issues appear correctable, they are substantial and affect the scientific reliability and reporting integrity of the manuscript.

Therefore, I recommend Major Revision before the manuscript can be considered for acceptance.

Once numerical inconsistencies, methodological clarifications, and reporting errors are fully corrected, the manuscript has the potential to make a meaningful contribution to the literature on adolescent metabolic risk in Latin America.

Sincerely,

Reviewer #5: This article by Curi-Quinto and collaborators is entitled : « Identification of a HOMA-IR cut-OFF point for cardiometabolis risk and modifiable risk factors in Peruvian adolescents ». This is the first revised version of a manuscript firstly sent last year to pone journal.

The aim of this article is to identify cut-off values from HOMA-IR assay in a cohort of Peruvian adolescents in order to propose an anticipated diagnosis for metabolic syndrome.

The Table 1 represents the percentile distribution of the HOMA-IR in the cohort studied and with criteria stratifications. Means from Z-score BMI/age revealed an expected link with overweight and obesity status.

The Table 2 is the repartition within the cohort studied following anthropometric and cardiometablic data. Similarly, BMI and BMI/Age showed a significant association with IR. Regarding cardiometabolic aspects, all parameters showed a significant association with IR but Diastolic blood pressure.

The Table 3 is a relative prediction of developing IR, following risk factors. Obesity and a high Fat mass index are significantly associated with IR.

The Table 4 is the multivariate regression model of the identified risk factors in the Peruvian adolescents population. This table only represents the female subgroup, adding low physical activity to the previous associations.

The Discussion part summarizes the results obtained with emphasis on the associations of BMI and cardiometabolic parameters. The independence of physical inactivity and high fat mass index should be tested and reviewed.

Figure 1 is a ROC curve to determine the optimal cut-off. This figure should be repositioned in the text as the starting element.

Figure 2 is the mean prevalence of the risk factors mentioned earlier within IR/not IR individuals. This figure should be repositioned in the text with a few words of introduction and purpose.

Some Typo :

Line 44 the end of the sentence is missing

Line 57 mails -> males

Line 75, 150 -> al. —> italic

Table 2 Cardio-metabolic —> Cardiometabolic

Line 288 y —> years old

And Sens :

Line 67 a reference is lacking linking hyperglycemia to hypertension at last

Line 76 « to the best of our knowledge » —> rephrase

Table 1 n = 371?

As a summary, authors should clarify their study, first with a revision of the order of the figures/tables and their introduction/description in the manuscript. And then with a sequential discussion of theses results. Several aspects could be compared to recent results from the literature (Lee et al. 2023, Lozano et al. 2022, Rocca-Nacion et al. 2022, Zelada et al. 2016).

**********

7. PLOS authors have the option to publish the peer review history of their article (what does this mean?). If published, this will include your full peer review and any attached files.

If you choose “no”, your identity will remain anonymous but your review may still be made public.

**Do you want your identity to be public for this peer review?**  For information about this choice, including consent withdrawal, please see our Privacy Policy.

Reviewer #1: No

Reviewer #2: No

Reviewer #5: No

**********

[NOTE: If reviewer comments were submitted as an attachment file, they will be attached to this email and accessible via the submission site. Please log into your account, locate the manuscript record, and check for the action link "View Attachments". If this link does not appear, there are no attachment files.]

To ensure your figures meet our technical requirements, please review our figure guidelines: https://journals.plos.org/plosone/s/figures

You may also use PLOS’s free figure tool, NAAS, to help you prepare publication quality figures: https://journals.plos.org/plosone/s/figures#loc-tools-for-figure-preparation.

NAAS will assess whether your figures meet our technical requirements by comparing each figure against our figure specifications.

Attachment Submitted filename: Reviewer Comments on PONE-D-25-02583_R1.docx

# Author comment: Author response to Decision Letter 2

**DOI:** 10.1371/journal.pone.0351139.r004

7 Apr 2026

Reviewer #2: Dear Editor,

I have carefully reviewed the revised version of manuscript PONE-D-25-02583R1 entitled “Identification of a HOMA-IR cut-off point for cardiometabolic risk and modifiable risk factors in Peruvian adolescents.”

The authors have made substantial efforts to address reviewer comments. The manuscript has improved in structure, clarity, and contextualization. The study addresses an important public health issue and provides regionally relevant data on insulin resistance and metabolic risk in adolescents.

However, several critical issues remain unresolved in the revised manuscript:

1. Persistent inconsistencies in reported sample size (349 vs 371 participants across sections and tables).

Thank you for your comment.

To clarify, all our main analyses were conducted using a sample of 371 participants. However, data on Fat Free Mass and Fat Free Mass Index were available for only 349 participants. It is important to note that the subset of 349 participants was used exclusively for a regression analysis (Table 3) and not for the main analyses, such as the identification of HOMA-IR.

Therefore, to avoid confusion, we have clarified this in the Study Design and Population section and have corrected the remaining inconsistencies throughout the manuscript.

From a total of 394 adolescents, we analyzed data of 371 participants that have complete data from infancy and adolescence. For fat-free mass (FFM) and fat-free mass index (FFMI), data were available for 349 participants; thus, related bivariate analyses used this subsample, while others used the full sample (n = 371).

See lines: 116 - 117

2. Implausible systolic blood pressure values are still present in Table 2.

Thank you for your observation. We acknowledge that there was a typographical error, which has now been corrected.

See lines: 223

3. Ethical section contains placeholder text that has not been properly revised.

Thank you for identifying the error. We have corrected it as follows:

The ethics committees of the Instituto de Investigación Nutricional (IIN), Lima-Peru, approved this study under the number 372-2017/CIEI-IIN. This study was conducted in accordance with the ethical principles of the Declaration of Helsinki. We obtained written informed consent from all participants prior to their inclusion as well as the assent from the adolescents.

See lines: 180-183

4. Ambiguity in the HOMA-IR calculation formula and unit reporting.

Thank you for the clarification. We have revised the wording of this section and have also added a relevant reference.

IR was estimated using the HOMA-IR, calculated as the product of fasting insulin (µU/mL) and fasting glucose (mmol/L), divided by 22.5. This index provides an indirect measure of insulin sensitivity based on fasting metabolic parameters [21].

See lines: 170-172

5. Overstatement of diagnostic performance (sensitivity/specificity described as “high”).

Thank you for the clarification. In the Discussion section, we have revised this statement to a more neutral wording and removed the overstatement, as follows:

In this study we identified a value of 3.90 as specific cut-off point for HOMA-IR that has a sensitivity of 72.4% and specificity of 75.4% for predicting MS in Peruvian adolescents with an average age of 14.5 years (AUC: 0.79; 95% CI 0.69–0.88).

See lines: 246-248

6. Minor but recurrent grammatical and reporting inconsistencies.

The manuscript has been thoroughly reviewed, and all errors have been corrected.

While these issues appear correctable, they are substantial and affect the scientific reliability and reporting integrity of the manuscript.

Therefore, I recommend Major Revision before the manuscript can be considered for acceptance.

Once numerical inconsistencies, methodological clarifications, and reporting errors are fully corrected, the manuscript has the potential to make a meaningful contribution to the literature on adolescent metabolic risk in Latin America.

Sincerely,

Reviewer #5:

This article by Curi-Quinto and collaborators is entitled : « Identification of a HOMA-IR cut-OFF point for cardiometabolis risk and modifiable risk factors in Peruvian adolescents ». This is the first revised version of a manuscript firstly sent last year to pone journal.

The aim of this article is to identify cut-off values from HOMA-IR assay in a cohort of Peruvian adolescents in order to propose an anticipated diagnosis for metabolic syndrome.

The Table 1 represents the percentile distribution of the HOMA-IR in the cohort studied and with criteria stratifications. Means from Z-score BMI/age revealed an expected link with overweight and obesity status.

The Table 2 is the repartition within the cohort studied following anthropometric and cardiometablic data. Similarly, BMI and BMI/Age showed a significant association with IR. Regarding cardiometabolic aspects, all parameters showed a significant association with IR but Diastolic blood pressure.

The Table 3 is a relative prediction of developing IR, following risk factors. Obesity and a high Fat mass index are significantly associated with IR.

The Table 4 is the multivariate regression model of the identified risk factors in the Peruvian adolescents population. This table only represents the female subgroup, adding low physical activity to the previous associations.

The Discussion part summarizes the results obtained with emphasis on the associations of BMI and cardiometabolic parameters. The independence of physical inactivity and high fat mass index should be tested and reviewed.

Thank you for the observation.

The methodology section has been expanded in response to your comments.

Figure 1 is a ROC curve to determine the optimal cut-off. This figure should be repositioned in the text as the starting element.

Thank you for the observation.

We have positioned Figure 1 as the first result in the Results section.

Results

The HOMA-IR cutoff identified was 3.9, yielding a sensitivity of 72.4% and a specificity of 75.4%. The area under the ROC curve was 0.79 (95% CI: 0.69–0.88) (Figure 1).

See lines: 195-196

Figure 2 is the mean prevalence of the risk factors mentioned earlier within IR/not IR individuals. This figure should be repositioned in the text with a few words of introduction and purpose.

We have positioned Figure 2 as the third result in the Results section, following Figure 1 and Table 1.

Some Typo :

Line 44 the end of the sentence is missing

Thank you for the observation.

The missing information has been added.

excess weight at infancy and FHCD.

See lines: 44

Line 57 mails -> males

Thank you for the observation.

The missing information has been added.

% in males and

See lines: 57

Line 75, 150 -> al. —> italic

Thank you for the observation.

The missing information has been added.

ped by Matthews et al. and this

loped by Kowalski et al, 1997 and

See lines: 75; 148

Table 2 Cardio-metabolic —> Cardiometabolic

Thank you for the observation.

The indicated issue has been corrected.

Line 288 y —> years old

Thank you for the observation.

The missing information has been added.

adolescents (7–14 years old); in both sexes

See lines: 268

And Sens :

Line 67 a reference is lacking linking hyperglycemia to hypertension at last

The reference has been added.

Sowers, J. R., Standley, P. R., Ram, J. L., Jacober, S., Simpson, L., & Rose, K. (1993). Hyperinsulinemia, insulin resistance, and hyperglycemia: contributing factors in the pathogenesis of hypertension and atherosclerosis. American journal of hypertension, 6(7 Pt 2), 260S–270S. https://doi.org/10.1093/ajh/6.7.260s

See lines: 68

Line 76 « to the best of our knowledge » —> rephrase

Thank you for the clarification. The wording of the sentence has been revised as follows:

Nonetheless, no studies in Peruvian adolescents have proposed specific cut-off points for HOMA-IR classification.

See lines: 79-80

Table 1 n = 371?

Yes, there was an error in the description of the total sample in the Methods section. The correct sample size is 371 adolescents. However, only the variables Fat Free Mass Index and Fat Mass Index in Tables 2 and 3 include data from 349 participants. We have clarified this in the Study Design and Population section to avoid confusion.

For fat-free mass (FFM) and fat-free mass index (FFMI), data were available for 349 participants; thus, related bivariate analyses used this subsample, while others used the full sample (n = 371).

See lines: 116-117

As a summary, authors should clarify their study, first with a revision of the order of the figures/tables and their introduction/description in the manuscript. And then with a sequential discussion of theses results. Several aspects could be compared to recent results from the literature (Lee et al. 2023, Lozano et al. 2022, Rocca-Nacion et al. 2022, Zelada et al. 2016).

Following your suggestion, we have carefully integrated the three recommended articles (Lee et al. 2023; Lozano et al., 2022; Rocca--Nación et al., 2022) into the manuscript. These references have been incorporated in a sequential and coherent manner within the Discussion, strengthening both the conceptual framework and the interpretation of our findings. As a result, the overall flow, depth, and clarity of the Discussion have been substantially improved.

See lines: 251-255; 257-261; 269-272

Attachment Submitted filename: Response Letter 07 04 2026.docx

# Peer review: Decision Letter 2

**DOI:** 10.1371/journal.pone.0351139.r005

**Authors:** Neftali Eduardo Antonio-Villa, Neftali Eduardo Antonio-Villa, Neftali Eduardo Antonio-Villa

11 May 2026

PONE-D-25-02583R2

IDENTIFICATION OF A HOMA-IR CUT-OFF POINT FOR CARDIOMETABOLIC RISK AND MODIFIABLE RISK FACTORS IN PERUVIAN ADOLESCENTS

PLOS One

Dear Dr. Curi-Quinto,

Thank you for submitting your manuscript to PLOS ONE. After careful consideration, we feel that it has merit but does not fully meet PLOS ONE’s publication criteria as it currently stands. Therefore, we invite you to submit a revised version of the manuscript that addresses the points raised during the review process.

Please submit your revised manuscript by Jun 25 2026 11:59PM. If you will need more time than this to complete your revisions, please reply to this message or contact the journal office at plosone@plos.org. When you're ready to submit your revision, log on to https://www.editorialmanager.com/pone/ and select the 'Submissions Needing Revision' folder to locate your manuscript file.

Please include the following items when submitting your revised manuscript:

- A letter that responds to each point raised by the academic editor and reviewer(s). You should upload this letter as a separate file labeled 'Response to Reviewers'.
- A marked-up copy of your manuscript that highlights changes made to the original version. You should upload this as a separate file labeled 'Revised Manuscript with Track Changes'.
- An unmarked version of your revised paper without tracked changes. You should upload this as a separate file labeled 'Manuscript'.

If you would like to make changes to your financial disclosure, please include your updated statement in your cover letter. Guidelines for resubmitting your figure files are available below the reviewer comments at the end of this letter.

If applicable, we recommend that you deposit your laboratory protocols in protocols.io to enhance the reproducibility of your results. Protocols.io assigns your protocol its own identifier (DOI) so that it can be cited independently in the future. For instructions see: https://journals.plos.org/plosone/s/submission-guidelines#loc-laboratory-protocols. Additionally, PLOS ONE offers an option for publishing peer-reviewed Lab Protocol articles, which describe protocols hosted on protocols.io. Read more information on sharing protocols at https://plos.org/protocols?utm_medium=editorial-email&utm_source=authorletters&utm_campaign=protocols.

As the corresponding author, your ORCID iD is verified in the submission system and will appear in the published article. PLOS supports the use of ORCID, and we encourage all coauthors to register for an ORCID iD and use it as well. Please encourage your coauthors to verify their ORCID iD within the submission system before final acceptance, as unverified ORCID iDs will not appear in the published article. *Only*  the individual author can complete the verification step; PLOS staff *cannot*  verify ORCID iDs on behalf of authors.

We look forward to receiving your revised manuscript.

Kind regards,

Neftali Eduardo Antonio-Villa, MD PhD

Academic Editor

PLOS One

Journal Requirements:

If the reviewer comments include a recommendation to cite specific previously published works, please review and evaluate these publications to determine whether they are relevant and should be cited. There is no requirement to cite these works unless the editor has indicated otherwise.

Please review your reference list to ensure that it is complete and correct. If you have cited papers that have been retracted, please include the rationale for doing so in the manuscript text, or remove these references and replace them with relevant current references. Any changes to the reference list should be mentioned in the rebuttal letter that accompanies your revised manuscript. If you need to cite a retracted article, indicate the article’s retracted status in the References list and also include a citation and full reference for the retraction notice.

Additional Editor Comments:

Please, address the final issues raised by the referees. Both of them, including this academic editor, agree that once the comments have been addressed, it can be suitable for publication.

[Note: HTML markup is below. Please do not edit.]

Reviewers' comments:

Reviewer's Responses to Questions

**Comments to the Author**

1. If the authors have adequately addressed your comments raised in a previous round of review and you feel that this manuscript is now acceptable for publication, you may indicate that here to bypass the “Comments to the Author” section, enter your conflict of interest statement in the “Confidential to Editor” section, and submit your "Accept" recommendation.

Reviewer #2: All comments have been addressed

Reviewer #5: All comments have been addressed

**********

2. Is the manuscript technically sound, and do the data support the conclusions?

The manuscript must describe a technically sound piece of scientific research with data that supports the conclusions. Experiments must have been conducted rigorously, with appropriate controls, replication, and sample sizes. The conclusions must be drawn appropriately based on the data presented.

Reviewer #2: Yes

Reviewer #5: Yes

**********

3. Has the statistical analysis been performed appropriately and rigorously?

Reviewer #2: Yes

Reviewer #5: Yes

**********

4. Have the authors made all data underlying the findings in their manuscript fully available?

The PLOS Data policy requires authors to make all data underlying the findings described in their manuscript fully available without restriction, with rare exception (please refer to the Data Availability Statement in the manuscript PDF file). The data should be provided as part of the manuscript or its supporting information, or deposited to a public repository. For example, in addition to summary statistics, the data points behind means, medians and variance measures should be available. If there are restrictions on publicly sharing data—e.g. participant privacy or use of data from a third party—those must be specified.

Reviewer #2: Yes

Reviewer #5: Yes

**********

5. Is the manuscript presented in an intelligible fashion and written in standard English?

PLOS ONE does not copyedit accepted manuscripts, so the language in submitted articles must be clear, correct, and unambiguous. Any typographical or grammatical errors should be corrected at revision, so please note any specific errors here.

Reviewer #2: Yes

Reviewer #5: Yes

**********

6. Review Comments to the Author

Please use the space provided to explain your answers to the questions above. You may also include additional comments for the author, including concerns about dual publication, research ethics, or publication ethics. (Please upload your review as an attachment if it exceeds 20,000 characters)

Reviewer #2: Before this manuscript can be accepted for publication, the authors must address the following minor but critical corrections:

Abstract Sample Size Inconsistency: In response to Reviewer #2 regarding sample size confusion, the authors clarified in the main text that the total sample is 371, with a sub-sample of 349 used specifically for FFM/FFMI analyses. Unfortunately, the authors neglected to update the Abstract, which still states: "A sample of 349 adolescents...". The Abstract must be updated to reflect the full cohort of 371.

Abstract Diagnostic Overstatement: The authors agreed to remove the subjective descriptor "high" when referring to sensitivity and specificity, modifying the Discussion accordingly. However, this overstatement was left intact in the Abstract, which still reads: "...with high sensitivity (72.4%) and specificity (75.4%).". This must be amended to match the neutral tone of the main text.

Scientific Typographical Error in Table 1: While the authors successfully corrected the HOMA-IR formula units in the Methods section (Lines 170-172) to µU/mL and mmol/L, the footnote for Table 1 contains a major scientific typo. It defines the calculation using fasting insulin in µU/L and fasting glucose in nmol/L. These units are incorrect for a divisor of 22.5 and must be corrected to match the main text.

Recommendation: Minor Revisions.

The core science, data, and conclusions of the study are sound. Once the authors align the Abstract and Table 1 footnotes with the corrections, they have already established in the main text, the manuscript will be highly suitable for publication and will make a valuable contribution to the understanding of adolescent metabolic risk in Latin America.

Reviewer #5: The article entitled « Identification of a HOMA-IR cut-off point for cardiometabolic risk and modifiable risk factors in Peruvian adolescents » by Curi-Quinto et al. is now in a second round of revision in Pone journal for a potential acceptance for publication.

To summarize, the aim of this study is to identify cut-off values associated with metabolic syndrome in a Peruvian adolescents population, with a distribution of HOMA-IR and a receiver operating characteristic (ROC). A multiple logistic regression analysis was applied concomitantly in order to identify the risk factors associated with insulin resistance.

Authors have diligently answered to the previous remarks from referees.

Please find enclosed few additional remarks regarding typos :

Line 192 a reference is lacking.

Police adjustments Line 162->167, Table 1, Line 501 -> 504.

**********

7. PLOS authors have the option to publish the peer review history of their article (what does this mean?). If published, this will include your full peer review and any attached files.

If you choose “no”, your identity will remain anonymous but your review may still be made public.

**Do you want your identity to be public for this peer review?**  For information about this choice, including consent withdrawal, please see our Privacy Policy.

Reviewer #2: No

Reviewer #5: No

**********

[NOTE: If reviewer comments were submitted as an attachment file, they will be attached to this email and accessible via the submission site. Please log into your account, locate the manuscript record, and check for the action link "View Attachments". If this link does not appear, there are no attachment files.]

To ensure your figures meet our technical requirements, please review our figure guidelines: https://journals.plos.org/plosone/s/figures

You may also use PLOS’s free figure tool, NAAS, to help you prepare publication quality figures: https://journals.plos.org/plosone/s/figures#loc-tools-for-figure-preparation.

NAAS will assess whether your figures meet our technical requirements by comparing each figure against our figure specifications.

Attachment Submitted filename: Reviewer Comments on PONE-D-25-02583_R2.pdf

# Author comment: Author response to Decision Letter 3

**DOI:** 10.1371/journal.pone.0351139.r006

13 May 2026

Reviewers' comments:

1. Abstract Sample Size Inconsistency: In response to Reviewer #2 regarding sample size confusion, the authors clarified in the main text that the total sample is 371, with a sub-sample of 349 used specifically for FFM/FFMI analyses. Unfortunately, the authors neglected to update the Abstract, which still states: "A sample of 349 adolescents...". The Abstract must be updated to reflect the full cohort of 371.

Thank you for the observation. We have corrected the error.

See lines: 33

2. Abstract Diagnostic Overstatement: The authors agreed to remove the subjective descriptor "high" when referring to sensitivity and specificity, modifying the Discussion accordingly. However, this overstatement was left intact in the Abstract, which still reads: "...with high sensitivity (72.4%) and specificity (75.4%).". This must be amended to match the neutral tone of the main text.

Thank you for the observation. The issue noted has been removed.

See lines: 40

3. Scientific Typographical Error in Table 1: While the authors successfully corrected the HOMA IR formula units in the Methods section (Lines 170-172) to µU/mL and mmol/L, the footnote for Table 1 contains a major scientific typo. It defines the calculation using fasting insulin in µU/L and fasting glucose in nmol/L. These units are incorrect for a divisor of 22.5 and must be corrected to match the main text.

Thank you for the observation. We have added the correct sample.

See table 1.

Attachment Submitted filename: Response Letter 11 05 26.docx

# Editor report: Decision Letter 3

**DOI:** 10.1371/journal.pone.0351139.r007

**Authors:** Neftali Eduardo Antonio-Villa, Neftali Eduardo Antonio-Villa, Neftali Eduardo Antonio-Villa, Neftali Eduardo Antonio-Villa

24 May 2026

IDENTIFICATION OF A HOMA-IR CUT-OFF POINT FOR CARDIOMETABOLIC RISK AND MODIFIABLE RISK FACTORS IN PERUVIAN ADOLESCENTS

PONE-D-25-02583R3

Dear Dr. Curi-Quinto,

We’re pleased to inform you that your manuscript has been judged scientifically suitable for publication and will be formally accepted for publication once it meets all outstanding technical requirements.

Within one week, you’ll receive an e-mail detailing the required amendments. When these have been addressed, you’ll receive a formal acceptance letter and your manuscript will be scheduled for publication.

An invoice will be generated when your article is formally accepted. Please note, if your institution has a publishing partnership with PLOS and your article meets the relevant criteria, all or part of your publication costs will be covered. Please make sure your user information is up-to-date by logging into Editorial Manager at Editorial Manager® and clicking the ‘Update My Information' link at the top of the page. For questions related to billing, please contact billing support.

If your institution or institutions have a press office, please notify them about your upcoming paper to help maximize its impact. If they’ll be preparing press materials, please inform our press team as soon as possible -- no later than 48 hours after receiving the formal acceptance. Your manuscript will remain under strict press embargo until 2 pm Eastern Time on the date of publication. For more information, please contact onepress@plos.org.

Kind regards,

Neftali Eduardo Antonio-Villa, MD PhD

Academic Editor

PLOS One

Additional Editor Comments (optional):

Dear authors. After carefully reviewing the new version of the manuscript, I agree that the corrections were Dear authors. After carefully reviewing the new version of the manuscript, I agree that the corrections are sufficient to endorse it for publication. Congratulations on the extensive work, as this paper could have useful and practical implications for clinical practice in Peru.

Reviewers' comments:

# Editor report: Acceptance letter

**DOI:** 10.1371/journal.pone.0351139.r008

**Authors:** Neftali Eduardo Antonio-Villa, Neftali Eduardo Antonio-Villa, Neftali Eduardo Antonio-Villa, Neftali Eduardo Antonio-Villa

PONE-D-25-02583R3

PLOS One

Dear Dr. Curi-Quinto,

I'm pleased to inform you that your manuscript has been deemed suitable for publication in PLOS One. Congratulations! Your manuscript is now being handed over to our production team.

At this stage, our production department will prepare your paper for publication. This includes ensuring the following:

* All references, tables, and figures are properly cited

* All relevant supporting information is included in the manuscript submission,

* There are no issues that prevent the paper from being properly typeset

You will receive further instructions from the production team, including instructions on how to review your proof when it is ready. Please keep in mind that we are working through a large volume of accepted articles, so please give us a few days to review your paper and let you know the next and final steps.

Lastly, if your institution or institutions have a press office, please let them know about your upcoming paper now to help maximize its impact. If they'll be preparing press materials, please inform our press team within the next 48 hours. Your manuscript will remain under strict press embargo until 2 pm Eastern Time on the date of publication. For more information, please contact onepress@plos.org.

You will receive an invoice from PLOS for your publication fee after your manuscript has reached the completed accept phase. If you receive an email requesting payment before acceptance or for any other service, this may be a phishing scheme. Learn how to identify phishing emails and protect your accounts at https://explore.plos.org/phishing.

If we can help with anything else, please email us at customercare@plos.org.

Thank you for submitting your work to PLOS ONE and supporting open access.

Kind regards,

PLOS ONE Editorial Office Staff

on behalf of

Dr. Neftali Eduardo Antonio-Villa

Academic Editor

PLOS One
